# 3-Mercaptopyruvate sulfur transferase is a protein persulfidase

**DOI:** 10.1038/s41589-022-01244-8

**Published:** 2023-02-02

**Authors:** Brandán Pedre, Deepti Talwar, Uladzimir Barayeu, Danny Schilling, Marcin Luzarowski, Mikolaj Sokolowski, Sebastian Glatt, Tobias P. Dick

**Affiliations:** 1 Division of Redox Regulation, DKFZ-ZMBH Alliance, German Cancer Research Center (DKFZ), Heidelberg, Germany; 2 Faculty of Biosciences, Heidelberg University, Heidelberg, Germany; 3 Core Facility for Mass Spectrometry and Proteomics, Centre for Molecular Biology at Heidelberg University (ZMBH), Heidelberg, Germany; 4 Max Planck Research Group, Malopolska Centre of Biotechnology, Jagiellonian University, Kraków, Poland

**Keywords:** Enzymes, Cell signalling, Post-translational modifications

## Abstract

Protein S-persulfidation (P-SSH) is recognized as a common posttranslational modification. It occurs under basal conditions and is often observed to be elevated under stress conditions. However, the mechanism(s) by which proteins are persulfidated inside cells have remained unclear. Here we report that 3-mercaptopyruvate sulfur transferase (MPST) engages in direct protein-to-protein transpersulfidation reactions beyond its previously known protein substrates thioredoxin and MOCS3/Uba4, associated with H_2_S generation and transfer RNA thiolation, respectively. We observe that depletion of MPST in human cells lowers overall intracellular protein persulfidation levels and identify a subset of proteins whose persulfidation depends on MPST. The predicted involvement of these proteins in the adaptation to stress responses supports the notion that MPST-dependent protein persulfidation promotes cytoprotective functions. The observation of MPST-independent protein persulfidation suggests that other protein persulfidases remain to be identified.

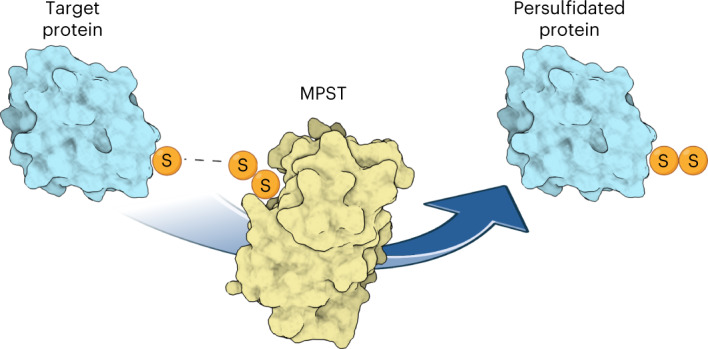

## Abstract (web summary)

Mercaptopyruvate sulfur transferase (MPST) is revealed as a protein persulfidase that acts directly on numerous and diverse target proteins, revealing potential origins of persulfidation as a common posttranslational modification.

## Main

Protein persulfidation (P-SSH) is now recognized as a posttranslational modification that naturally occurs inside cells across all domains of life^1^. Proteomic analyses have shown that persulfidation affects a large number of functionally diverse proteins^1–3^. However, the physiological role and importance of protein persulfidation remains to be fully understood. Protein persulfidation exists under homeostatic conditions and is often observed to increase under conditions of oxidative stress^1,2,4^. Similar to other oxidative protein thiol modifications, persulfidation may activate or inactivate individual proteins, thus potentially adapting protein function to changing conditions^5–7^. In addition, protein persulfidation may protect protein thiols against irreversible oxidation^8,9^. However, it is not known how proteins are actually persulfidated inside cells.

Potential nonenzymatic mechanisms of protein persulfidation have been discussed previously^10^. First, some protein thiols may react with H_2_O_2_ to become sulfenylated, and then with H_2_S to become persulfidated. Second, some protein disulfide bonds may react with H_2_S to generate a persulfide. However, both reactions are slow^11^ and unlikely to explain the fact that so many proteins can be detected in the persulfidated state, even under apparent nonstress conditions. We recently noted that conditions that oxidize protein thiols to sulfenic acids also oxidize any resulting hydropersulfides to perthiosulfonic acids, at least in vitro^12^. It is thus difficult to see how H_2_O_2_ can trigger the formation of hydropersulfides without immediately oxidizing these. Third, low molecular-weight (LMW) persulfides, such as GSSH and Cys-SSH, known to be generated inside cells^13^, have been proposed to transfer single sulfur atoms to thiols^14^, but in fact are not observed to engage in transpersulfidation^10^. Given these and other considerations, it has long been speculated that posttranslational protein persulfidation is facilitated enzymatically by one or more sulfur transferases^15^.

In this study we investigated 3-mercaptopyruvate sulfur transferase (MPST), a sulfur transferase that so far has been mainly associated with H_2_S generation and transfer RNA thiolation. MPST desulfurates 3-mercaptopyruvate (3MP), a product of cysteine catabolism, to generate pyruvate and an enzyme-bound persulfide. In contrast to small molecule persulfides, the MPST-bound persulfide is capable of transferring its outer sulfur atom to thiol acceptors, facilitated by a specialized steric and electronic environment^16^. Until now, two protein substrates of MPST have been known. MPST persulfidates thioredoxin in the context of H_2_S generation^17^, and MOCS3/Uba4 for subsequent tRNA thiolation and protein urmylation^18,19^.

However, it has been observed previously that overexpression of MPST increases intracellular ‘bound’ sulfane sulfur (S^0^) content, potentially indicating a direct role for MPST in general protein persulfidation^20–23^. Nonetheless, this possibility has not been tested so far. In this study, we present experimental evidence supporting the notion that MPST acts as a protein persulfidase and makes a major contribution to overall protein persulfidation.

Starting out with purified proteins in vitro, we observed that redox-sensitive green fluorescent protein (roGFP2), a protein with two thiol groups on its surface, acts as a highly efficient sulfur acceptor for MPST. We also observed bovine serum albumin (BSA) accepting sulfur from MPST. These observations indicated to us that MPST may have a broad protein persulfidating activity. We confirmed that transfer of sulfur from MPST to roGFP2 also takes place inside living yeast cells and that roGFP2 oxidation is due to a direct protein-to-protein transpersulfidation reaction. Further in vitro experiments indicated that MPST is not a major producer of inorganic polysulfides, again supporting the notion that direct transpersulfidation is the predominant mode of MPST-mediated protein persulfidation. We then showed that depletion of MPST in human cells significantly lowers overall cellular protein persulfidation levels. We identified a set of 64 target proteins whose persulfidation largely depends on MPST. Taken together, we conclude that MPST has the intrinsic ability to persulfidate a broad range of target proteins under physiologically relevant conditions. The predicted involvement of these proteins in the adaptation to stress responses likely explains previously reported phenotypes of MPST deletion in model organisms.

## Results

### MPST-roGFP2 couples 3MP desulfuration to roGFP2 oxidation

Following the design principle of previous roGFP2-based biosensors^24^, we engineered an MPST-roGFP2 fusion protein, Tum1-roGFP2, based on the yeast MPST homolog thiouridine modifying protein 1 (Tum1). We hypothesized that 3MP desulfuration by MPST should lead to roGFP2 disulfide formation through a mechanism that involves three consecutive steps, namely (1) formation of an MPST-bound persulfide (Pyr-SH (≡ 3MP) + MPST-SH → Pyr-H + MPST-SSH), (2) transpersulfidation of roGFP2 (MPST-SSH + roGFP2(-SH)_2_ → MPST-SH + roGFP2(-SH)(-SSH)) and (3) roGFP2 intramolecular disulfide bond formation coupled to H_2_S release (roGFP2(-SH)(-SSH) → roGFP2(S-S) + H_2_S).

**Fig. 1:**
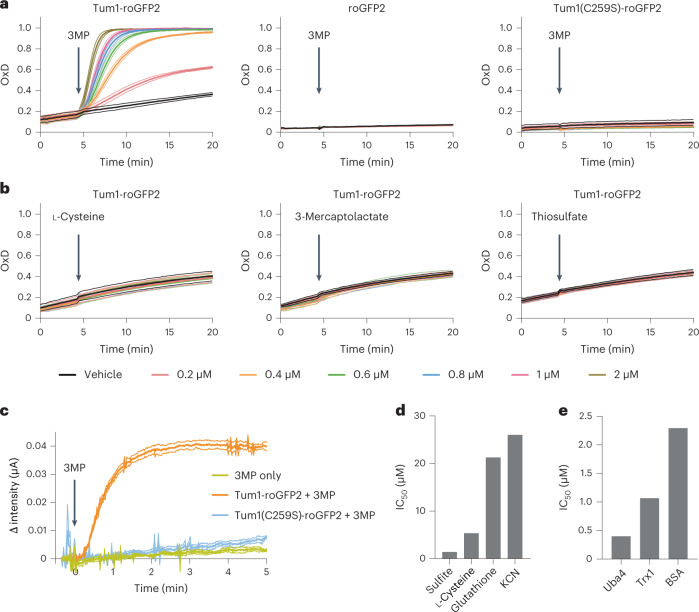
Tum1-roGFP2 couples 3MP desulfuration to roGFP2 oxidation, releasing H_2_S in the process. **a**, Degree of oxidation (OxD) of 100 nM Tum1-roGFP2 (left panel), roGFP2 (center panel) and Tum1(C259S)-roGFP2 (right panel), in response to increasing 3MP concentrations. **b**, Degree of oxidation of 100 nM Tum1-roGFP2, in response to increasing concentrations of l-cysteine (left panel), 3-mercaptolactate (center panel) and thiosulfate (right panel). **c**, H_2_S release from 2.5 μM Tum1-roGFP2 on addition of 50 μM 3MP, as measured by an H_2_S-selective electrode. **d**, The half-maximum inhibitory concentration (IC_50_) values for LMW compounds acting as Tum1 sulfur acceptors. **e**, IC_50_ values for proteins acting as Tum1 sulfur acceptors. All data are based on *n* = 3 independent experiments. Source data

**Extended Data Fig. 1:**
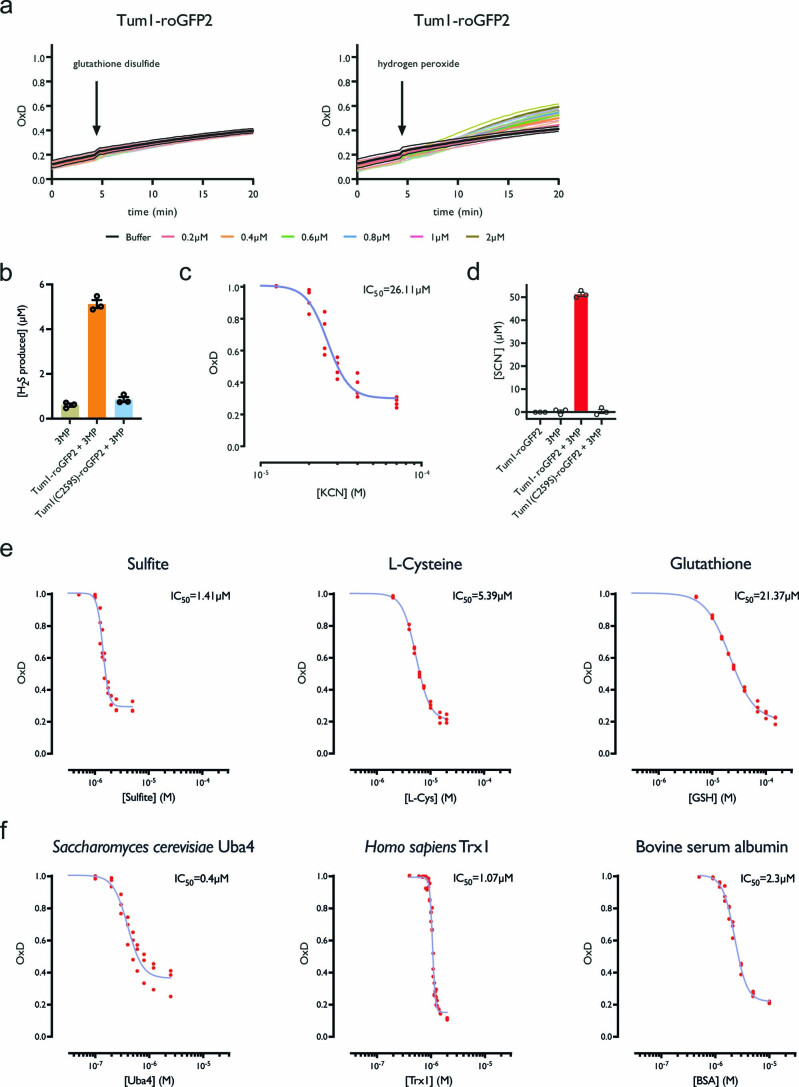
Comparison of sulfur acceptors for MPST. (**a**) Degree of oxidation (OxD) of Tum1-roGFP2 in response to increasing glutathione disulfide (left panel) or hydrogen peroxide concentrations (right panel). (**b**) Concentrations of H_2_S as measured 5 min after addition of 3MP. Data are presented as mean and individual values (n = 3 independent experiments) +/- SEM. (**c**) Competition of 3MP-dependent Tum1-roGFP2 oxidation (OxD) by increasing concentrations of potassium cyanide (KCN). (**d**) Catalytically active Tum1-roGFP2 converts cyanide to thiocyanate (SCN), in a 3MP- dependent manner. Data are presented as mean and individual values (n = 3 independent experiments) +/- SEM. (**e**) Competition of 3MP-dependent Tum1-roGFP2 oxidation (OxD) by increasing concentrations of sulfite (left panel), l-cysteine (center panel), and glutathione (right panel). (**f**) Competition of 3MP-dependent Tum1-roGFP2 oxidation (OxD) by increasing concentrations of *S. cerevisiae* Uba4 (left panel), *H. sapiens* thioredoxin 1 (Trx1) (center panel), and bovine serum albumin (right panel). All data are based on n = 3 independent experiments. Source data

To investigate the recombinant fusion protein, we compared it to roGFP2 and to a mutant fusion protein, Tum1(C259S)-roGFP2, in which the active site cysteine of Tum1 is replaced by serine. First, we tested its response to 3MP in vitro. The intact fusion protein, but neither roGFP2 nor Tum1(C259S)-roGFP2, was oxidized in response to 3MP (Fig. 1a), thus demonstrating chemical communication between the active site of the MPST domain and the dithiol-disulfide site of roGFP2. Of note, Tum1-roGFP2 did not show any response to either l-cysteine (l-Cys) or 3-mercaptolactate (Fig. 1b, left and middle panels). These compounds are chemically related molecules upstream and downstream of 3MP in metabolism. Likewise, Tum1-roGFP2 did not respond to thiosulfate (Fig. 1b, right panel), the preferred substrate of thiosulfate sulfur transferases, which are the members of the rhodanese family most closely related to the MPSTs. Tum1-roGFP2 was nonresponsive toward GSSG (oxidized glutathione) and showed only a marginal response to H_2_O_2_ (Extended Data Fig. 1a). Considering the mechanism proposed above, 3MP-mediated roGFP2 oxidation is expected to be accompanied by the generation of H_2_S. Indeed, the reaction of 3MP with Tum1-roGFP2, but not with Tum1(C259S)-roGFP2, led to the release of H_2_S (Fig. 1c and Extended Data Fig. 1b).

### Sulfite and proteins are preferred sulfur acceptors for MPST

**Extended Data Fig. 2:**
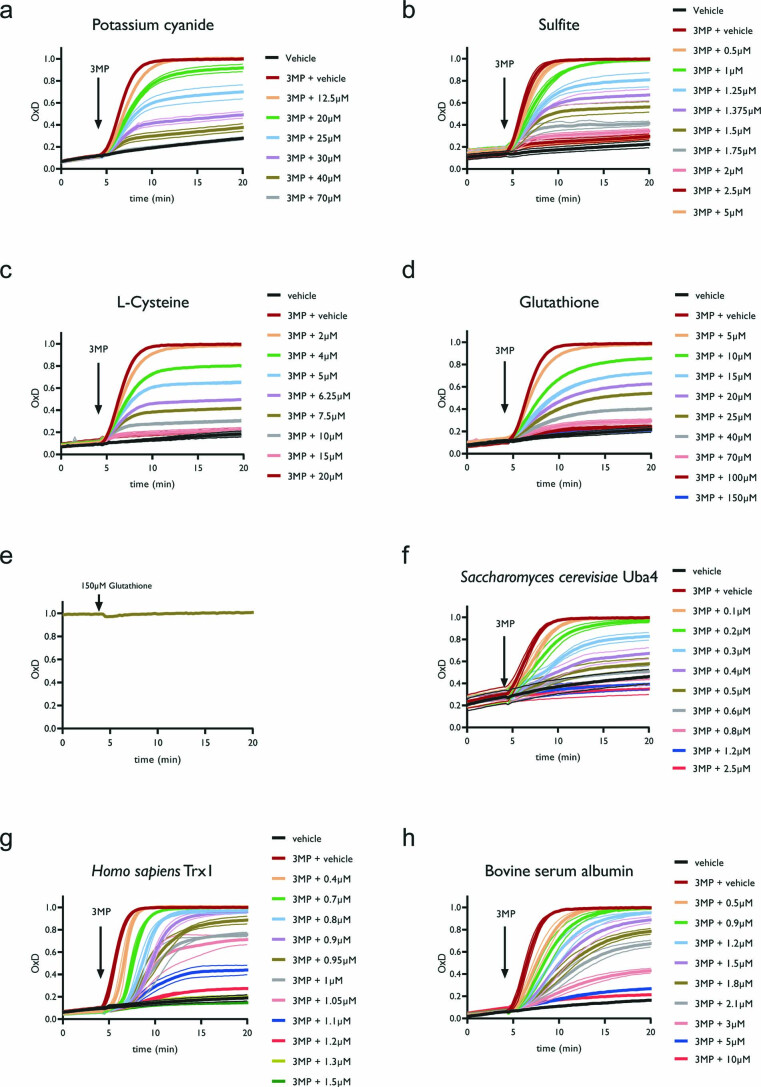
Competition between roGFP2 and other sulfur acceptors. (**a**) Competition of 3MP-dependent Tum1-roGFP2 oxidation (OxD) by increasing concentrations of potassium cyanide (KCN). (**b**) Competition of 3MP-dependent Tum1-roGFP2 oxidation (OxD) by increasing concentrations of sulfite. (**c**) Competition of 3MP-dependent Tum1-roGFP2 oxidation (OxD) by increasing concentrations of L-cysteine. (**d**) Competition of 3MP-dependent Tum1-roGFP2 oxidation (OxD) by increasing concentrations of glutathione. (**e**) Lack of reduction of oxidized Tum1-roGFP2 upon the addition of 150 μM glutathione. (**f**) Competition of 3MP-dependent Tum1-roGFP2 oxidation (OxD) by increasing concentrations of *S. cerevisiae* Uba4. (**g**) Competition of 3MP-dependent Tum1-roGFP2 oxidation (OxD) by increasing concentrations of *H. sapiens* thioredoxin 1 (Trx1). (**h**) Competition of 3MP-dependent Tum1-roGFP2 oxidation (OxD) by increasing concentrations of bovine serum albumin. All data are based on n = 3 independent experiments, except (e) (n = 1). Source data

Having established that Tum1-fused roGFP2 acts as a sulfur acceptor for Tum1, we then investigated the ability of other potential sulfur acceptors to compete with roGFP2 for taking over the 3MP-derived sulfur from the Tum1 domain. Therefore, we added increasing concentrations of acceptor candidates and monitored their impact on 3MP-dependent roGFP2 oxidation. As a proof of concept, we first used cyanide, a well-established sulfur acceptor for MPST^25^. As expected, increasing amounts of cyanide suppressed 3MP-dependent roGFP2 oxidation in a concentration-dependent fashion (Extended Data Figs. 1c and 2a), forming thiocyanate as the product (Extended Data Fig. 1d). Comparing several small molecule sulfur acceptors (Fig. 1d), we identified sulfite as an outstanding competitor (Extended Data Fig. 1e, left panel and Extended Data Fig. 2b), while l-Cys and glutathione (GSH) were less efficient (Extended Data Fig. 1e, middle and right panels and Extended Data Fig. 2c,d). GSH acted as a bona fide competitor of sulfur transfer, as it was not able to reduce the already oxidized fusion protein (Extended Data Fig. 2e). Comparing protein acceptors (Fig. 1e), we found that the known MPST/Tum1 substrates yeast Uba4 and human Trx1 are very good competitors (Extended Data Fig. 1f, left and middle panels and Extended Data Fig. 2f,g). However, BSA, which is not a natural substrate of MPST/Tum1, was almost as effective (Extended Data Fig. 1f, right panel and Extended Data Fig. 2h), suggesting that MPST has a general ability to persulfidate accessible thiol groups on other proteins. In summary, we found that the Tum1-roGFP2 fusion protein couples 3MP desulfuration to roGFP2 thiol oxidation through the mediacy of a transferable sulfur atom, with high specificity and efficiency. Competition experiments further revealed that protein clients are the most efficient sulfur acceptors, with the exception of sulfite. Obviously, MPST does not only sulfurate previously characterized protein substrates (Trx, Uba4), but also other proteins not normally encountered by MPST in its natural context (roGFP2, BSA).

### Response of the MPST-roGFP2 fusion protein in yeast

**Fig. 2:**
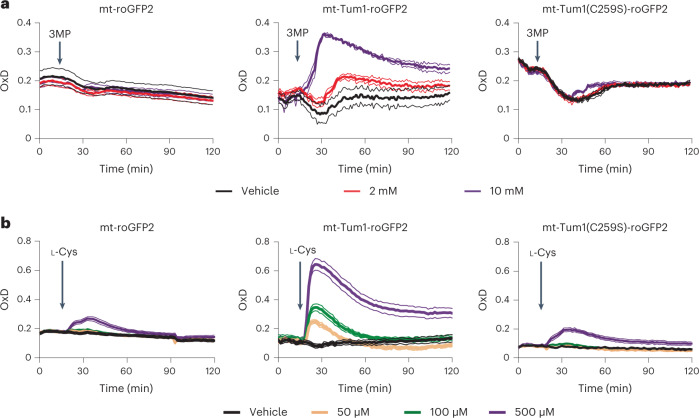
Mitochondrial Tum1-roGFP2 responds to 3MP and l-Cys. **a**, Response of roGFP2 (left), Tum1-roGFP2 (center) and Tum1(C259S)-roGFP2 (right), expressed in the mitochondrial matrix (mt), to exogenously added 3MP. **b**, Response of roGFP2 (left), Tum1-roGFP2 (center) and Tum1(C259S)-roGFP2 (right), expressed in the mitochondrial matrix (mt), to exogenously added l-Cys. Data are based on *n* = 3 independent experiments, except for Tum1-roGFP2 + 3MP (*n* = 2). Source data

**Fig. 3:**
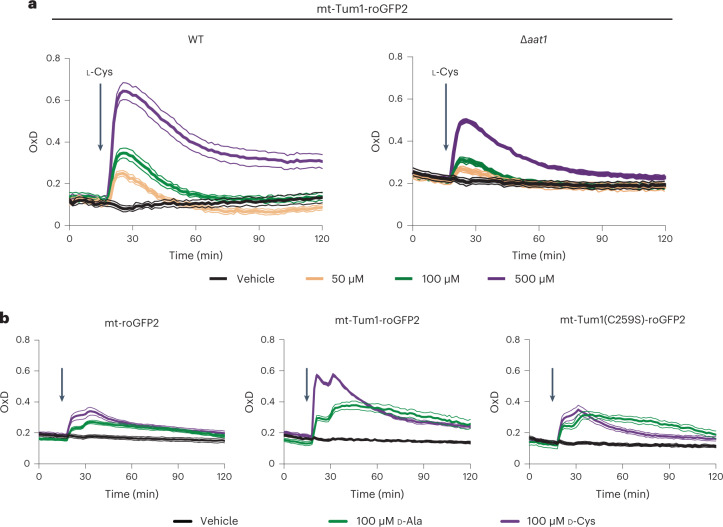
Mitochondrial Tum1-roGFP2 responds to endogenously produced 3MP. **a**, Response of mitochondrial Tum1-roGFP2 to l-Cys in the parental strain (WT, left panel) and in a strain lacking mitochondrial cysteine transaminase (Δ*aat1*, right panel). **b**, Response of mitochondrial probes roGFP2 (left), Tum1-roGFP2 (center) and Tum1(C259S)-roGFP2 (right) to exogenously added d-Cys (purple) or d-Ala (green), in a strain expressing mitochondrial DAAO. All data are based on *n* = 3 independent experiments. Source data

**Extended Data Fig. 3:**
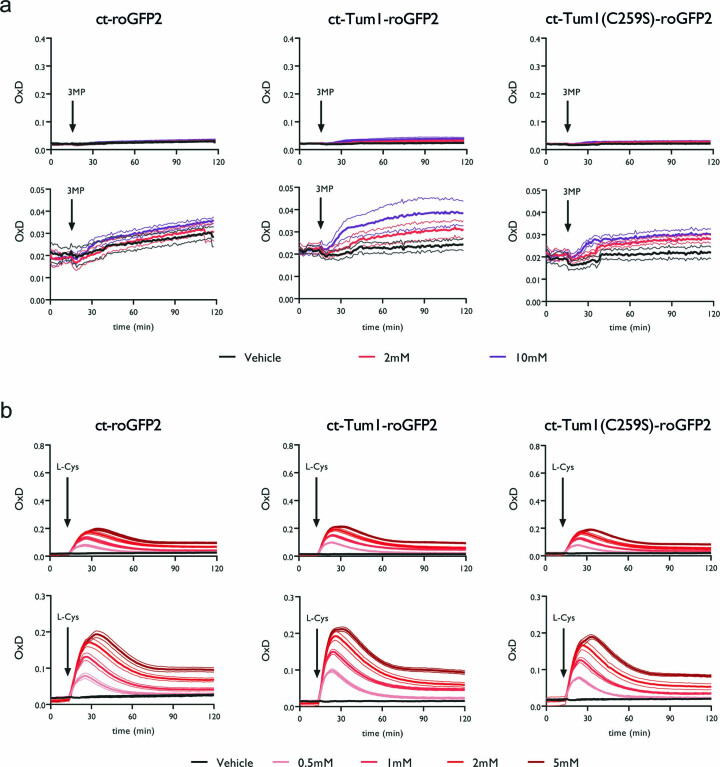
Response of cytosolic probes to 3MP and l-Cys. (**a**) Response of roGFP2 (left panels), Tum1-roGFP2 (center panels) and Tum1(C259S)-roGFP2 (right panels), expressed in the cytosol (ct), to exogenously added 3MP. The lower panels show the same curves on a smaller y axis scale. (**b**) Response of roGFP2 (left panels), Tum1-roGFP2 (center panels) and Tum1(C259S)-roGFP2 (right panels), expressed in the cytosol (ct), to exogenously added l-Cys. The lower panels show the same curves on a smaller y axis scale. All data are based on n = 3 independent experiments. Source data

**Extended Data Fig. 4:**
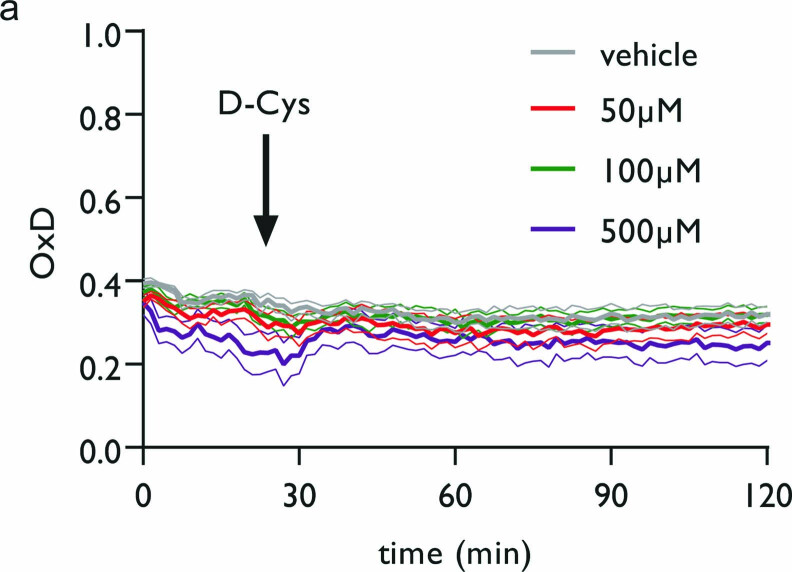
Response of mitochondrial Tum1-roGFP2 to d-Cys. (**a**) Mitochondrial Tum1-roGFP2 does not respond to exogenously added d-cysteine (d-Cys) when expressed in cells lacking D-amino acid oxidase. Data are based on n = 3 independent experiments. Source data

Having characterized MPST-dependent roGFP2 oxidation in vitro, we expressed the fusion protein in the cytosol and mitochondrial matrix of yeast cells, along with the two controls, roGFP2 and Tum1(C259S)-roGFP2. First, we tested the response of the probes to exogenously supplied 3MP. The mitochondrial probe responded markedly (Fig. 2a, middle panel), while the cytosolic one responded only weakly (Extended Data Fig. 3a, middle panels). Next, we tested the response to exogenously supplied l-Cys, the metabolic precursor of 3MP. l-Cys gave rise to a stronger probe response than 3MP, presumably due to more efficient cellular uptake of l-Cys and its subsequent intracellular conversion to 3MP. Again, we observed a prominent response in the mitochondria (Fig. 2b, middle panel), but only a weak one in the cytosol (Extended Data Fig. 3b, middle panels). Based on these findings, we further investigated the response of the mitochondrial probe to exogenous l-Cys. To confirm that the observed response reflects the endogenous generation of 3MP, we deleted *aat1*, the gene encoding the mitochondrially located transaminase that is known to convert l-Cys to 3MP. Indeed, the response of the mitochondrial probe to l-Cys was blunted in the absence of *aat1* (Fig. 3a). We then directly generated 3MP inside mitochondria, by expressing mitochondrially targeted d-amino acid oxidase (DAAO), which converts d-Cys into 3MP. Since DAAO also produces H_2_O_2_, which may cause roGFP2 oxidation, we also tested the probe response to d-alanine (d-Ala), which also produces H_2_O_2_, but not 3MP. Indeed, we observed that mitochondrial Tum1-roGFP2 responded more strongly to d-Cys than to d-Ala, unlike roGFP2 or Tum1(C259S)-roGFP2 (Fig. 3b). The observed response was DAAO-dependent, as d-Cys did not trigger a response in the absence of DAAO (Extended Data Fig. 4a). In conclusion, the Tum1-roGFP2 fusion protein, expressed in yeast mitochondria, responds to 3MP, which is endogenously produced from l-Cys.

### MPST sulfurates roGFP2 independently of forced proximity

**Fig. 4:**
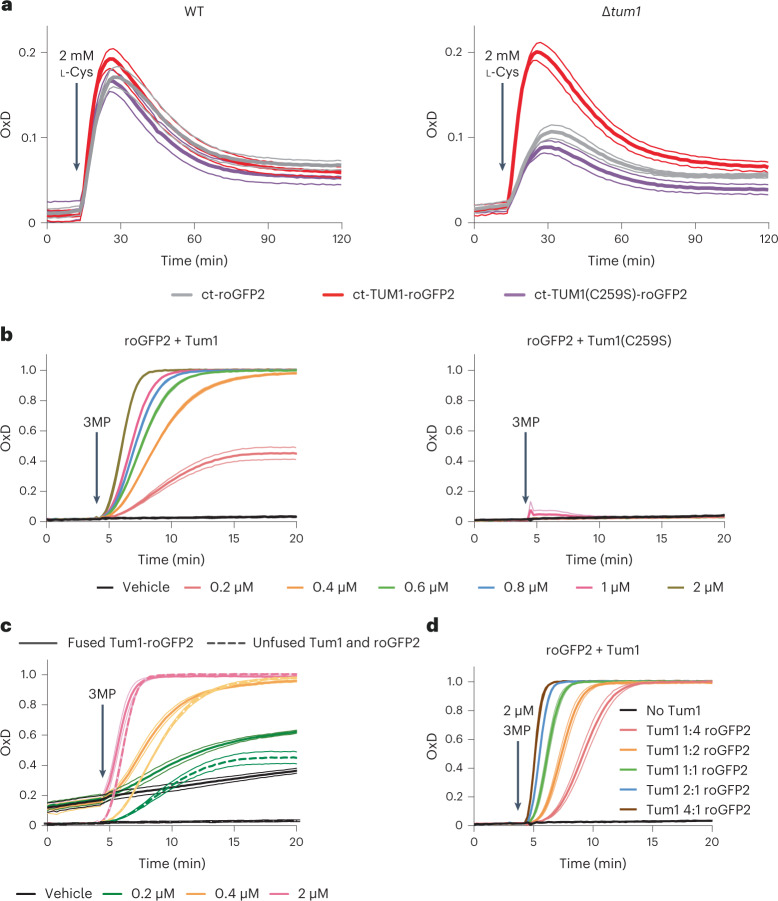
Tum1 oxidizes roGFP2 independently of forced proximity. **a**, Response of cytosolic (ct) Tum1-roGFP2 to l-Cys in the parental (WT, left) and Δ*tum1* strain (right). **b**, In vitro response of roGFP2 (100 nM) to increasing 3MP concentrations in the presence of an equimolar amount of 100 nM wild-type (left panel) or mutant Tum1 (right panel). **c**, In vitro response of the Tum1-roGFP2 fusion protein (100 nM) to 3MP (solid lines) in comparison to the response of an equimolar mixture of 100 nM roGFP2 and 100 nM Tum1 (dashed lines). **d**, In vitro response of roGFP2 (100 nM) to 3MP in the presence of increasing concentrations of Tum1. All data are based on *n* = 3 independent experiments. Source data

While mitochondrial Tum1-roGFP2 responded much better than mitochondrial roGFP2 to 3MP (or the 3MP precursor l-Cys), this was not the case in the cytosol, where we could not detect differences in responsiveness. Notably, roGFP2 responded almost as strongly to l-Cys as did Tum1-roGFP2 (Extended Data Fig. 3b, left and middle panels). We hypothesized that this may be due to the fact that endogenous Tum1 is predominantly located in the cytosol^26^. Hence, the response of unfused roGFP2 in the cytosol may be facilitated by interactions with endogenous Tum1, thus making the fusion of Tum1 dispensable. To investigate the influence of endogenous Tum1 we compared the reactivity of the probe in wild-type and Δ*tum1* cells. We found that the response of unfused roGFP2 in the cytosol largely depends on the presence of endogenous Tum1 (Fig. 4a). This experiment shows that Tum1 efficiently transfers sulfur to roGFP2 when both proteins are expressed separately in the same cellular compartment. Using purified proteins we confirmed that free Tum1 is efficient in oxidizing free roGFP2 on addition of 3MP (Fig. 4b). A direct side-by-side comparison revealed that an equimolar mixture of free proteins is as efficient in facilitating roGFP2 oxidation as the corresponding Tum1-roGFP2 fusion protein (Fig. 4c). The rate of roGFP2 oxidation was further enhanced by increasing Tum1 concentration (Fig. 4d). These results indicate that Tum1 efficiently oxidizes roGFP2 irrespectively of an artificially enforced proximity.

**Fig. 5:**
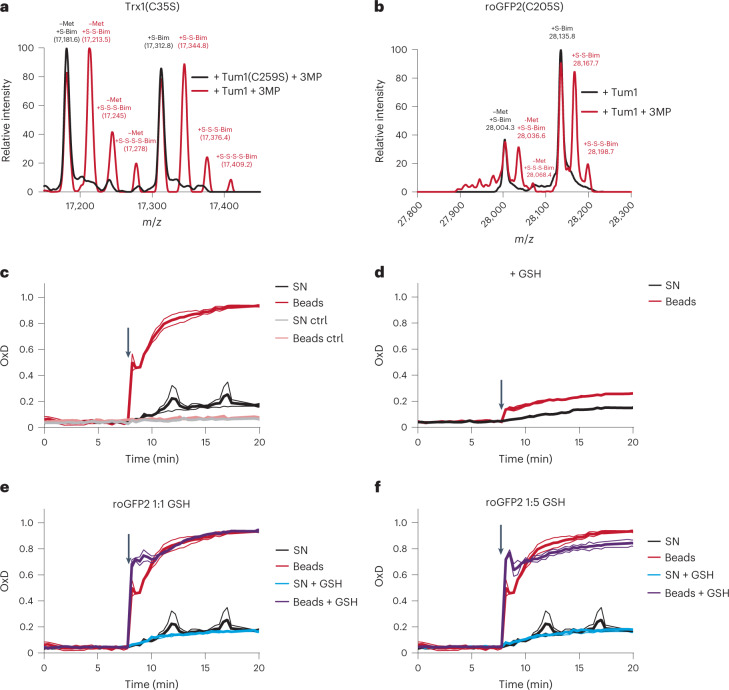
Tum1 directly persulfidates other proteins. **a**,**b**, Mass spectra of *H. sapiens* Trx1(C35S) (**a**) and roGFP2(C205S) (**b**) exposed to Tum1 and 3MP (red curve), or to an inactive Tum1 system (black curve). *n* = 1. **c**, Oxidation of roGFP2 (420 nM) by immobilized Tum1-SSH, in the absence of LMW compounds (beads; red line), or by the corresponding supernatant (SN; black line) of the reaction between immobilized Tum1 (2 µM) and 3MP (100 µM), that is, in the absence of Tum1-SSH. Control (ctrl) experiments were performed in absence of 3MP. *n* = 2 independent experiments. **d**, The same experiment as in **c**, but with the initial reaction between immobilized Tum1 (2 µM) and 3MP (100 µM) conducted in the presence of GSH (100 µM), thus diminishing formation of MPST-SSH. *n* = 2 independent experiments. **e**, Oxidation of roGFP2 (420 nM) by immobilized Tum1-SSH in the presence of GSH (beads + GSH; purple line), or by the corresponding supernatant in the presence of GSH (SN + GSH; blue line). Left panel: 420 nM GSH. Right panel: 2,100 nM GSH. The curves obtained in **c** (beads and SN in the absence of GSH; red and black lines, respectively) are included for direct comparison. *n* = 2 independent experiments. Source data

**Extended Data Fig. 5:**
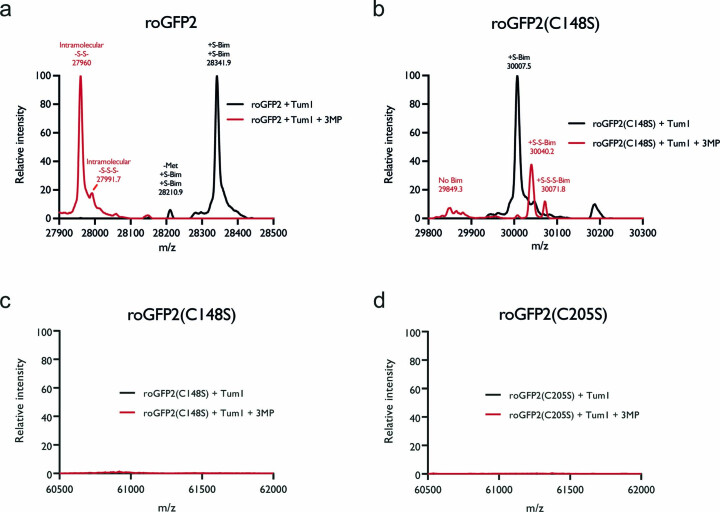
Tum1 oxidizes roGFP2 by transpersulfidation, not disulfide exchange. (**a-b**) Mass spectra of roGFP2 (**a**) and roGFP2(C148S) (**b**) exposed to Tum1 in the presence (red curves) or absence (black curves) of 3MP. n = 1 experiment. (**c-d**) Mass spectra of roGFP2(C148S) (**c**) and roGFP2(C205S) (**d**) in the m/z = 60500-62000 region. The lack of signals indicates the absence of mixed disulfide conjugates between Tum1 and roGFP2. n = 1 experiment. Source data

The above proposed mechanism predicts that Tum1 transpersulfidates roGFP2 on one of its two surface thiols. To directly detect protein persulfidation we performed whole protein mass spectrometry, using monobromobimane to trap protein persulfides. As a positive control, we incubated Tum1 with a mono-thiol Trx1 mutant (TrxC35S) and added 3MP. In the presence of active Tum1, but not in the presence of mutated Tum1(C259S), we observed the addition of 1 to 3 sulfur masses to Trx(C35S) (corresponding to Trx-SSH, Trx-SSSH and Trx-SSSSH) (Fig. 5a). This result confirmed that Tum1 transfers sulfur to Trx1. We then asked whether Tum1 would also transfer sulfur to roGFP2. First, we incubated Tum1 with normal (dithiol) roGFP2. On addition of 3MP, roGFP2 was oxidized to the disulfide form, and we also observed the appearance of a trisulfide (Extended Data Fig. 5a). To capture the persulfide intermediate of the reaction, we then repeated the experiment with the two mono-thiol variants of roGFP2 (C148S and C205S). In both cases, we detected the formation of the corresponding persulfide (Fig. 5b and Extended Data Fig. 5b). We did not detect a mixed disulfide between Tum1 and roGFP2 in any of these experiments (Extended Data Fig. 5c,d). These observations directly confirmed that MPST can transfer sulfur not only to its dedicated protein substrates, but also to an arbitrary nonnatural substrate (roGFP2), suggesting that MPST acts as a general protein persulfidase, potentially contributing to overall intracellular protein persulfidation. In addition, the outcome of our experiments provides unequivocal proof that roGFP2 is indeed oxidized through transpersulfidation and not by a thiol-disulfide exchange reaction with MPST.

### Sulfur transfer does not involve small molecule intermediates

**Extended Data Fig. 6:**
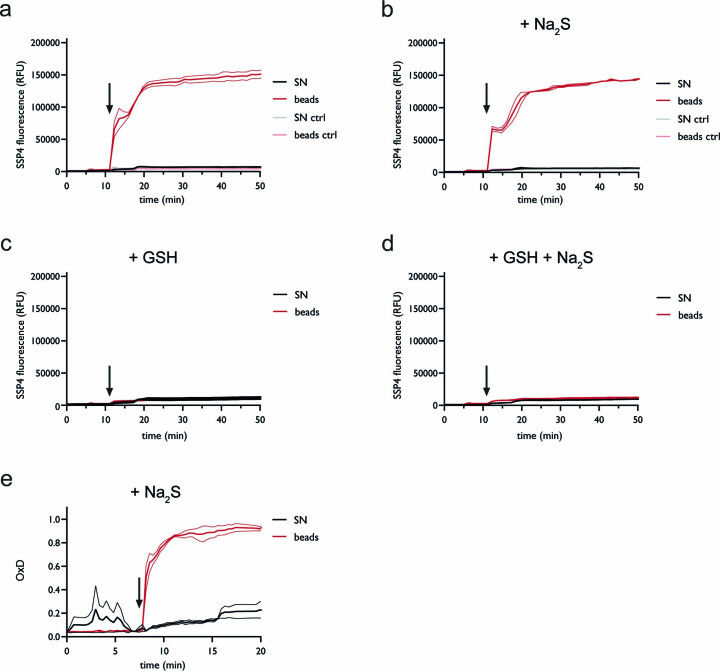
MPST-bound sulfane sulfur is not released into the supernatant. (**a-d**) Reactivity of SSP4 towards immobilized Tum1-SSH (beads, red lines), or towards the corresponding supernatant (SN, black lines) of the reaction between immobilized Tum1 and 3MP, in the absence (**a**), or presence of Na_2_S (10 µM) (**b**), in the presence of GSH (100 µM) (**c**), and in the presence of both GSH (100 µM) and Na_2_S (10 µM) (**d**). Control (ctrl) experiments were performed in absence of 3MP. Data are based on n = 2 independent experiments. (**e**) Oxidation of roGFP2 by immobilized Tum1-SSH (beads, red line), or by the corresponding supernatant (SN, black line) of the reaction of immobilized Tum1 and 3MP, performed in the presence of Na_2_S (10 µM). Data are based on n = 2 independent experiments. Source data

While the above-described experiments indicated direct protein-to-protein transpersulfidation, they did not rule out an alternative possibility. Previous studies have suggested that MPST can generate inorganic polysulfides (H_2_S_2_, H_2_S_3_)^22,23^, which in principle could facilitate target protein persulfidation (for example, P-SH + H_2_S_2_ → P-SSH + H_2_S). To find out whether MPST-mediated protein persulfidation is direct (that is, protein-to-protein) or indirect (that is, through soluble LMW polysulfides), we devised an experiment that allowed us to distinguish between the two possible mechanisms. To this end, we immobilized recombinantly expressed and purified streptavidin-binding-peptide (SBP)-tagged Tum1 on streptavidin agarose (SA) beads and incubated it with 3MP to generate bead-bound Tum1-SSH. The supernatant was separated from the beads and the beads were washed to ensure the absence of small molecules. Beads and supernatants were then tested separately and side-by-side. The reaction with the S^0^ probe SSP4 revealed that >95% of S^0^ is associated with the beads (Extended Data Fig. 6a), suggesting that generation of inorganic polysulfides by MPST is a minor process. The additional presence of H_2_S did not make a difference (Extended Data Fig. 6b), suggesting that even at supraphysiological concentration (10 µM) H_2_S is not an efficient sulfur acceptor for MPST and hence does not promote H_2_S_2_ generation. As expected, the presence of a large (100-fold) molar excess of GSH (during the incubation of bead-bound MPST with 3MP) largely abolished bead-associated SSP4 reactivity (Extended Data Fig. 6c,d), as S^0^ is transferred to and reduced by GSH. Next, we tested the reaction of roGFP2 with beads and supernatants. About 90% of roGFP2 oxidizing activity was associated with the beads (Fig. 5c), again showing that small molecule products make a minor contribution to the observed roGFP2 oxidation. Moreover, bead-associated oxidation was much faster, indicating a kinetic advantage of direct protein-to-protein transpersulfidation. Again, the presence of additional H_2_S did not make a difference (Extended Data Fig. 6e). Similar to the SSP4 experiment, the presence of a large excess of GSH (during the incubation of bead-bound MPST with 3MP) diminished the yield of Tum1-SSH, and therefore of roGFP2 oxidation (Fig. 5d). In sum, these experiments confirmed that the observed oxidation of roGFP2 is predominantly the result of direct transpersulfidation. LMW species potentially generated by MPST (polysulfides) appear to play a minor role, if any.

As shown above, a roughly 200-fold excess of GSH over Tum1-roGFP2 inhibited roGFP2 oxidation by 50% (Extended Data Fig. 1e, right panel), and a 50-fold excess of GSH over Tum1 diminished Tum1-SSH availability for roGFP2 by roughly 90% (Fig. 5d). This raised the question whether GSH is likely to outcompete MPST-mediated protein persulfidation under intracellular conditions. GSH is often considered to outnumber all other thiols inside cells. However, the pool of accessible protein thiols is at least as large as the GSH pool^27,28^. To directly test whether GSH limits the protein persulfidase activity of MPST when protein thiols are as abundant as GSH, we first prepared and washed Tum1-SSH, and then monitored the direct competition of GSH and roGFP2 at molar ratios of 1:1 and 5:1. Neither ratio had a notable influence on roGFP2 oxidation (Fig. 5e). This suggests that under intracellular conditions, when MPST is as likely to meet a protein thiol as a LMW thiol, protein persulfidation is not substantially limited by the presence of GSH.

### MPST contributes to overall protein persulfidation

**Fig. 6:**
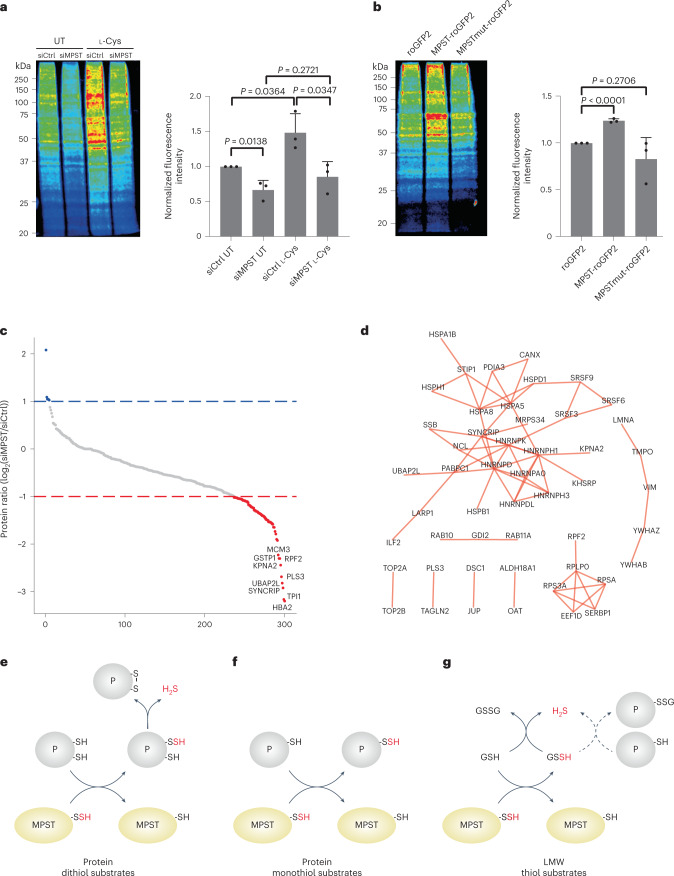
MPST contributes to global protein persulfidation. **a**, Overall persulfidation levels in HEK293 MSR cells before and after depletion of MPST (left panel). Cells were treated with 5 mM l-Cys for 30 min or were left untreated (UT). Relative persulfidation levels are indicated by Coomassie-normalized fluorescence intensity (right panel). Data are presented as mean and individual values (*n* = 3 biologically independent experiments) ± s.e.m. Statistical analysis based on a two-tailed unpaired *t*-test. **b**, Overall persulfidation levels in HEK293 MSR cells ectopically overexpressing roGFP2, MPST-roGFP2 or MPST(C248S)-roGFP2 (MPSTmut-roGFP2) (left panel). Relative persulfidation levels are indicated by Coomassie-normalized fluorescence intensity (right panel). Data are presented as mean and individual values (*n* = 3 biologically independent experiments) ± s.e.m. Statistical analysis based on a two-tailed unpaired *t*-test. **c**, Influence of MPST depletion on the persulfidation of individual proteins. Proteins depleted by at least twofold in MPST-depleted cells are marked in red. **d**, Interaction analysis of candidate MPST target proteins. Edges represent experimentally supported protein–protein interactions (confidence score >0.4) acquired from the STRING database^50^. The graph was generated with Cytoscape^51^. **e**–**g**, Summary of MPST-driven transpersulfidation. The MPST-bound persulfide (MPST-SSH) sulfurates thiol-containing molecules, the outcome depending on the type of acceptor. **e**, Sulfur transfer to proteins (P) with vicinal dithiols (roGFP2, Trx1) generates a protein disulfide and releases H_2_S. **f**, Sulfur transfer to protein monothiols leads to longer-lived protein persulfides. **g**, Sulfur transfer to GSH generates GSSH, which releases H_2_S to generate GSSG or (dotted lines) to glutathionylate proteins. Source data

**Extended Data Fig. 7:**
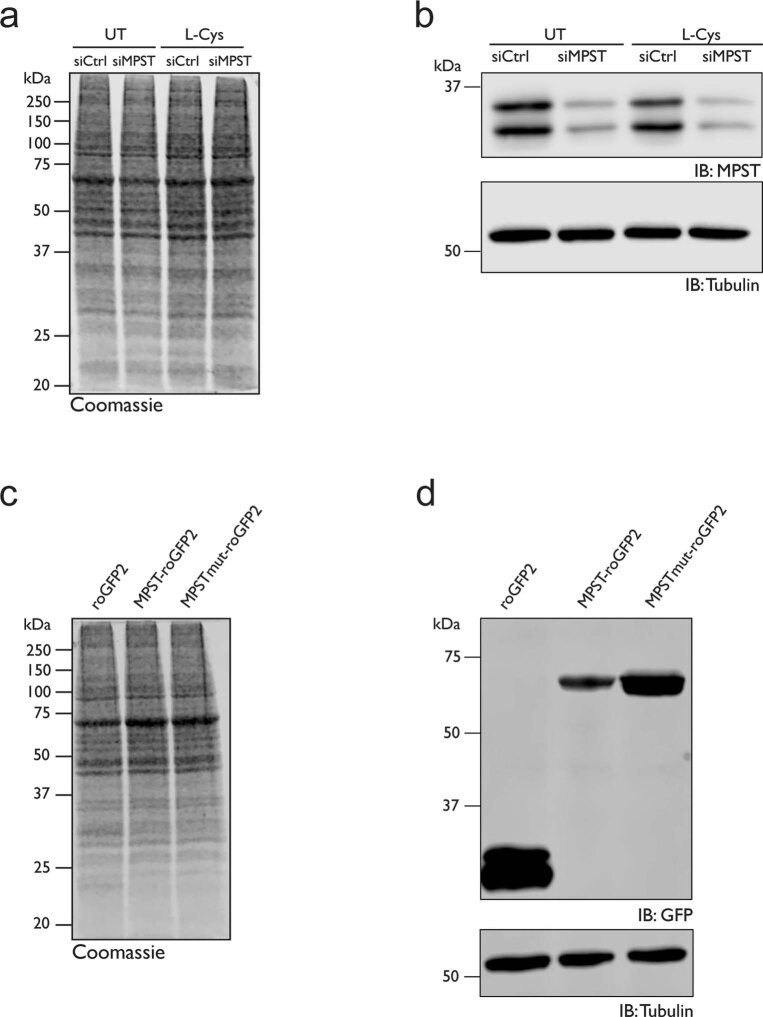
Depletion and overexpression of MPST. (**a**) Loading control for the experiment shown in main Fig. 6a. (**b**) Depletion of MPST in HEK293 MSR cells, as demonstrated by anti-MPST immunoblotting. (**c**) Loading control for the experiment shown in main Fig. 6b. (**d**) Overexpression of roGFP2 (left), MPST-roGFP2 (center), and MPST(C259S)-roGFP2 (MPSTmut-roGFP2, right) in HEK293 MSR cells, as demonstrated by anti-GFP immunoblotting. All data are based on n ≥ 3 replicates. Source data

Following our observation that Tum1 was capable of persulfidating several thiol-containing proteins and facilitated intracellular roGFP2 persulfidation when coexpressed in the same cellular compartment, we then asked whether Tum1 contributes to overall intracellular protein persulfidation. To this end, we monitored protein persulfidation using the dimedone switch assay^1^. Initially we tried to apply this technique to yeast cells either expressing or lacking Tum1. However, despite intense effort, we were unable to obtain reliable and reproducible results. We were also not able to obtain reproducible results for yeast cells expressing or lacking cystathionine-γ-lyase, reported previously^1^, suggesting technical limitations in applying the technique to yeast. We therefore decided to test the validity of our previous findings by monitoring overall protein persulfidation in human cells subjected to MPST depletion. In contrast to yeast cells, the results obtained for human cells were highly reproducible. We found baseline protein persulfidation levels to be lower in MPST-depleted cells (Fig. 6a and Extended Data Figs. 7a,b). Moreover, provisioning of extra l-Cys to the medium increased persulfidation in MPST-proficient, but not in MPST-depleted cells (Fig. 6a and Extended Data Figs. 7a,b). Conversely, ectopic overexpression of MPST, but not of the catalytically inactive MPST mutant, increased intracellular persulfidation levels (Fig. 6b and Extended Data Figs. 7c,d). To identify individual target proteins of MPST, we again performed the dimedone switch assay, this time coupling persulfides to biotin instead of a fluorescent dye. We compared the abundance of biotinylated proteins in mock-depleted and MPST-depleted cells using mass spectrometry-based label free quantitation and identified 64 proteins that were substantially depleted on MPST depletion (Fig. 6c). Many of these proteins are directly or indirectly involved in stress responses. Interaction analysis further suggests selectivity toward particular processes and protein families (Fig. 6d). In conclusion, we find that the MPST expression level (in connection with cysteine availability) is a major contributing factor to overall protein persulfidation in human cells.

## Discussion

Protein persulfidation has been recognized as a common posttranslational modification of physiological relevance, but the actual mechanism of protein persulfidation has remained elusive.

Previously, several nonenzymatic mechanisms of protein persulfidation have been proposed. One idea is that LMW persulfides (GSSH or Cys-SSH) react with protein thiols to generate protein persulfides (for example, P-SH + GSSH → P-SSH + GSH)^14^. However, this is not observed and also seems chemically implausible, as thiols can be expected to attack the inner sulfur atom of the persulfide to release H_2_S as the leaving group^10^. Thus, the reaction with LMW persulfides should rather lead to protein *S*-glutathionylation and *S*-cysteinylation, respectively (for example, P-SH + GSSH → P-SSG + H_2_S). A second idea is that protein persulfidation occurs through a two-step reaction with H_2_O_2_ and H_2_S: first, oxidation of the thiol to the sulfenic acid (P-SH + H_2_O_2_ → P-SOH + H_2_O), followed by condensation with H_2_S to form the persulfide (P-SOH + H_2_S → P-SSH + H_2_O)^1,11^. This mechanism is chemically feasible^11^ and may occur under conditions of substantial oxidative stress (for example, when cells are exposed to high amounts of oxidants)^1^. However, it is unlikely to explain basal protein persulfidation or to be of general relevance, because H_2_O_2_ and H_2_S are naturally produced at nanomolar concentrations, and most protein thiols are not very reactive toward H_2_O_2_ (*k* ≅ 1–10 M^−1^ s^−1^)^29^. A third idea is the cleavage of protein disulfide bonds by H_2_S. However, the intrinsic reactivity of HS^−^ toward disulfides is even one order of magnitude lower than that of thiolates^11^. A fourth idea is that inorganic LMW polysulfides, in particular H_2_S_2_, could be responsible for driving protein persulfidation (P-SH + H_2_S_2_ → P-SSH + H_2_S). This reaction is known to be efficient and has been exploited to persulfidate proteins in vitro^12^. However, it remains unclear to which extent H_2_S_2_ is formed inside cells and whether it contributes to protein persulfidation.

In this paper, we identified an enzymatic pathway that contributes to overall intracellular protein persulfidation. We found that the sulfur transferase MPST is highly efficient in persulfidating diverse proteins, both in vitro and inside cells. To investigate the mechanism by which MPST mediates protein persulfidation, we primarily used the model target protein roGFP2. Like the physiological MPST target protein Trx1, roGFP2 has two vicinal thiols, which means that persulfidation triggers subsequent disulfide bond formation (roGFP2(-SH)(-SSH) → roGFP2(S-S) + H_2_S). Using single Cys mutants of roGFP2 we directly detected MPST-mediated roGFP2 persulfidation by mass spectrometry (Fig. 5b and Extended Data Fig. 5b), confirming that MPST-mediated roGFP2 oxidation is indeed due to sulfur transfer (MPST-SSH + roGFP2(-SH)_2_ → MPST-SH + roGFP2(-SH)(-SSH) → MPST-SH + roGFP2(S-S) + H_2_S) (Fig. 6e) and not due to disulfide bond exchange (MPST-SSH + roGFP2(-SH)_2_ → MPST-S-S-roGFP2(-SH) + H_2_S → MPST-SH + roGFP2(S-S) + H_2_S).

Previously, it has been suggested that MPST is capable of generating inorganic polysulfides, including H_2_S_2_ (ref. ^22^). We therefore asked whether the observed sulfur transfer from MPST to roGFP2 is indeed a direct one or whether it may be mediated through a diffusible LMW inorganic polysulfide. We found that MPST-SSH, in the absence of LMW products, is highly efficient in persulfidating roGFP2, while LMW products, in the absence of MPST-SSH, were barely able to oxidize roGFP2. This outcome was not influenced by the presence of H_2_S, suggesting that H_2_S is not an efficient sulfur acceptor for MPST (MPST-SSH + H_2_S → MPST-SH + H_2_S_2_). Together, these results confirmed that the MPST-bound persulfide is capable of direct sulfur transfer to protein thiols (Fig. 6f). This finding is in line with previous structural and mechanistic insights: The MPST active site allows an attack on the outer sulfur atom while shielding the inner sulfur atom of the enzyme-bound persulfide against nucleophilic attack^16^. This explains why MPST-SSH transpersulfidates other thiols while LMW persulfides (GSSH, Cys-SSH) do not.

Despite the preference of MPST for protein substrates, a large excess of GSH can outcompete roGFP2 oxidation (MPST-SSH + GSH + GSH → MPST-SH + GSSH + GSH → MPST-SH + GSSG + H_2_S) (Fig. 6g). Inhibition of roGFP2 oxidation by a large excess of GSH has also been observed for MPSTs from *Arabidopsis thaliana*^30^. This raises the question whether GSH can be expected to inhibit MPST-mediated protein persulfidation in the cellular context. Although GSH is the most abundant LMW thiol in eukaryotic cells (roughly 2–10 mM), the pool of accessible protein thiols seems to be at least as large as the GSH pool. The protein thiol pool has been estimated to constitute up to 70% of cellular thiol content^27^ and to be roughly 25-fold more concentrated than GSH in mitochondria^28^. A direct competition experiment showed that an equimolar concentration of GSH does not affect MPST-mediated protein persulfidation and that a moderate (fivefold) molar excess of GSH affected protein persulfidation only slightly (Fig. 5e). Thus, it seems that MPST-mediated sulfur transfer to other proteins can take place under typical intracellular conditions, that is, even in the presence of millimolar GSH concentrations.

We started out by investigating an MPST-roGFP2 fusion protein because we initially assumed that enforced proximity is needed to allow for efficient sulfur transfer between the two proteins. This expectation was based on previous experience with other roGFP2 fusion proteins. In particular, close proximity enables the function of H_2_O_2_ probes in which roGFP2 is fused to and oxidized by a thiol peroxidase. For example, the fusion between roGFP2 and the thiol peroxidase Orp1 responds much faster to H_2_O_2_ than the corresponding equimolar mixture of the individual protein domains^31^. Moreover, inside cells the sulfenic acid and disulfide intermediates formed on the thiol peroxidase are likely to react with other thiols (for example, GSH) if the target protein (roGFP2) is not kept in close proximity. In contrast to thiol peroxidases, we found MPST to be efficient in oxidizing roGFP2 regardless of being fused, even when both proteins were coexpressed as independent proteins in the yeast cytosol. This suggests that inside the cell the MPST-bound persulfide is not rapidly intercepted by LMW thiols, but long-lived enough to be transferred to other proteins. This is also in line with our observation that proteins are generally better MPST substrates than LMW thiols. In the MPST crystal structure^32^, the active site persulfide appears to be relatively inaccessible. This may explain why it is only moderately reactive toward GSH and other LMW substrates, with the exception of sulfite. Notably, the active site is located at the interface between two rhodanese domains, potentially suggesting that this cleft can be opened up by protein–protein interactions, thus allowing for protein-to-protein transpersulfidation.

When we depleted MPST in human cells overall intracellular protein persulfidation was clearly diminished, although not totally abolished. This indicates that MPST facilitates a substantial part of overall protein persulfidation, and also suggests that there are proteins persulfidated by other sulfur transferases or other mechanisms. It is conceivable that other rhodanese family sulfur transferases (such as thiosulfate sulfur transferase) can also act as protein persulfidases. Our findings do not exclude the possibility that a fraction of protein persulfidation is caused by nonenzymatic mechanisms, as discussed above. It may be speculated that nonselective nonenzymatic protein persulfidation serves to protect thiols against hyperoxidation, while enzymatic protein persulfidation is more selective and serves to adapt protein functions.

Using a proteomics approach, we identified 64 proteins whose persulfidation levels were clearly decreased on MPST depletion. All of them are predicted to be located in the (nucleo)cytoplasm or in the mitochondria, conforming to the known intracellular distribution of MPST. Notably, more than half of the identified proteins have a known nuclear localization. The only seeming exceptions are calnexin and ERp57 (PDIA3), which are best known as ER-resident proteins. However, the transmembrane protein calnexin has a cysteine-containing cytoplasmic tail^33^ and ERp57 has previously been detected outside the ER^34^, thus potentially explaining their identification as MPST target proteins.

Notably, three quarters of the proteins identified here as MPST targets were previously identified as persulfidated in a mouse ‘persulfidome’ study^35^. For example, the three proteins whose persulfidation was most strongly affected by MPST depletion, alpha hemoglobin (HBA2/HBA1), triosephosphate isomerase (TPI1) and heterogeneous nuclear ribonucleoprotein Q (hnRNP Q, SYNCRIP), were previously found to be persulfidated in mouse kidney, liver, skeletal muscle and heart^35^. In addition, TPI1 was found to be persulfidated in HEK293 cells^36^, erythrocytes^1^ and A549 cells^37^.

Across a broad range of organisms, MPST knockouts have been found to be more sensitive to oxidative stress and/or to exhibit higher levels of endogenous oxidant levels^16^. We therefore wondered whether the MPST target proteins identified here may have roles in stress adaptation. Indeed, for many of them a connection to oxidant or electrophile stress adaptation has been reported or suggested. For example, alpha hemoglobin has been found to be upregulated in nonerythrocytes under oxidative stress conditions and to have a cytoprotective function^38,39^. TPI has been observed to undergo thiol redox modifications during stress responses^40–42^, potentially indicating a role in stress adaptation. hnRNP Q has been reported to regulate NADPH oxidase 2 expression in macrophages^43^. Glutathione S-transferase π1 (GSTP1), also previously observed to be persulfidated^35,36^, may depend on persulfidation for some of its detoxifying functions^44^. It is also interesting to note that four proteins identified here (ERp57, tubulin beta-3, nucleolin and hnRNP K), were reported to form a complex whose upregulation was associated with increased chemoresistance^45^.

Interactome analysis further suggests that MPST has a preference to persulfidate hnRNPs and proteins involved in proteostasis, including various heat shock proteins. Cysteine residues of hnRNPs are often found oxidized^46^ and are known to be susceptible to electrophile adduction, especially within RNA recognition motifs^47^. Specifically, the thiol redox state of hnRNP K has been reported to modulate the heat shock response^48^, supporting the idea that hnRNP thiol modifications can play adaptive regulatory roles. Another cluster of MPST target proteins is organized around vimentin. Vimentin is sensitive to electrophiles and oxidants and its redox state reorganizes the vimentin network in response to stresses^49^.

In conclusion, many proteins we identified as MPST target proteins appear to be regulated by thiol modifications and in turn regulate processes that adapt cells to stressful conditions. Thus, it is plausible that persulfidation of these proteins by MPST contributes to cytoprotection. Therefore, the recognition of MPST as a protein persulfidase may help to explain the observed phenotypes of MPST deficiency, namely diminished cytoprotection.

## Methods

### Reagents

All reagents used in this study are listed in Supplementary Table 1.

### Expression constructs

Yeast expression constructs are based on a sequence encoding codon-optimized roGFP2 and a 5xGGSGG linker repeat^52^. The sequence encoding of *Saccharomyces* *cerevisiae* Tum1 was obtained by PCR-amplification from yeast genomic DNA. The coding sequence for the fusion protein was assembled in the p415TEF vector, using the NEBuilder HiFi DNA Assembly Master Mix (New England Biolabs). For targeting to the mitochondrial matrix, constructs additionally include the N-terminal mitochondrial targeting sequence from F_0_-ATPase subunit 9 (Su9) from *Neurospora crassa* (p415TEF Su9roGFP2). Cysteines of Tum1 (C259) and roGFP2 (C148 and C205) were changed to serines using the Quikchange Site-Directed Mutagenesis Kit (Agilent). The DAAO coding sequence was PCR-amplified from pC1-CMV-DAAO-NES (ref. ^53^), kindly provided by V. Belousov. All plasmids and primers used in this study are listed in Supplementary Tables 2 and 3, respectively. The Tum1-linker-roGFP2 construct was recloned into the pET-His-SUMO vector (Thermo Fisher Scientific) using the NEBuilder HiFi DNA Assembly Master Mix (New England Biolabs).

### Recombinant protein expression and purification

Expression and purification of recombinant roGFP2-His and *H. sapiens* Trx1 was performed as described previously^54,55^. His-SUMO-Tum1-roGFP2 and His-SUMO-Tum1 were expressed in *Escherichia coli* BL21(DE3). Luria-Bertani (LB) medium was inoculated with a single colony, incubated overnight and then diluted 1:100 in terrific broth. The culture was grown at 37 °C with shaking, and on reaching an optical density (OD) absorbance of A_600nm_ = 0.6, expression was induced by adding 1 mM isopropyl-β-d-thiogalactopyranoside (IPTG). Following overnight incubation at room temperature, cells were collected by centrifugation at 4,000*g* for 15 min at 4 °C. After one freeze–thaw cycle, the cells were resuspended in B-PER Bacterial Protein Extraction Reagent (Thermo Fisher Scientific) supplemented with 0.5 mM dithiothreitol (DTT), 5 mM imidazole, EDTA-free Protease Inhibitor Cocktail (cOmplete, Roche) and Benzonase Nuclease (Merck Millipore). The lysate was clarified by centrifugation at 18,000*g* for 45 min at 4 °C, filtered through a 0.45 mm filter and added to Ni^2+^-Sepharose beads equilibrated with 50 mM Tris, pH 8, 500 mM NaCl, 5 mM imidazole and 0.5 mM DTT. The lysate-bead suspension was rotated for 30 min at 4 °C and subsequently packed into a 5-ml Pierce centrifuge column (Thermo Fisher Scientific). After washing with 50 mM Tris, pH 8, 500 mM NaCl, 5 mM imidazole and 0.5 mM DTT, the protein was eluted by increasing the imidazole concentration in 50 mM steps. Imidazole was removed by overnight dialysis in 50 mM Tris, pH 8, 200 mM NaCl, 0.5 mM DTT at 4 °C and the His-SUMO tag was cleaved by digestion with 2 U per 100 µg of PreScission Protease, overnight at 4 °C. The His-SUMO tag and PreScission Protease were removed by passing the reaction mixture through a Ni^2+^-Sepharose column and a GST-sepharose column, preequilibrated with the same buffer. The protein was finally purified by size-exclusion chromatography using a Superdex 75 Increase 10/300 GL column equilibrated with 20 mM Tris, pH 8, 150 mM NaCl, 1 mM EDTA and 0.5 mM DTT, using the ÄKTA Pure fast protein liquid chromatography (LC) system (Cytiva). Finally, the purified protein was flash-frozen in liquid nitrogen and stored at −80 °C.

SBP-His-SUMO-Tum1 was expressed in *E. coli* BL21(DE3). LB medium was inoculated with a single colony, incubated overnight and then diluted 1:100 in terrific broth. The culture was grown at 37 °C with shaking, and on reaching an OD absorbance of A_600nm_ = 0.7 expression was induced by adding 0.5 mM IPTG. Following overnight incubation at 37 °C, cells were gathered by centrifugation at 4,000*g* for 15 min at 4 °C. After one freeze–thaw cycle, the cells were resuspended in B-PER Bacterial Protein Extraction Reagent (Thermo Fisher Scientific) supplemented with 0.5 mM DTT, EDTA-free Protease Inhibitor Cocktail (cOmplete, Roche) and Benzonase Nuclease (Merck Millipore). The lysate was clarified by centrifugation at 18,000*g* for 45 min at 4 °C, filtered through a 0.45 mm filter and streptavidin sepharose high performance beads (SA beads; GE Healthcare) were added for affinity purification of protein. After 1 h incubation of the lysate-bead suspension at 4 °C, SA beads were washed three times with the wash buffer (50 mM Tris, pH 8, 300 mM NaCl, 1 mM EDTA and 0.5 mM DTT). The protein was eluted after incubation of SA beads in wash buffer containing 4 mM biotin for 20 min at 4 °C. Biotin was removed by overnight dialysis in 50 mM Tris, pH 8, 250 mM NaCl and 0.5 mM DTT at 4 °C. The purified protein was flash-frozen in liquid nitrogen and stored at −80 °C.

*S. cerevisiae* Uba4 was expressed from the pRARE plasmid in *E. coli* BL21 (DE3) grown in LB media at 18 °C and induced overnight with 0.5 M IPTG. The bacterial pellet was resuspended in lysis buffer (30 mM HEPES pH 8.0; 300 mM NaCl; 20 mM imidazole; 0.15% TX-100; 10 mM MgSO_4_; 1 mM 2-mercaptoethanol; 10 mg ml^−1^ DNase; 1 mg ml^−1^ lysozyme; 10% glycerol and a cocktail of protease inhibitors) and lysed to homogeneity using a high-pressure homogenizer (Emulsiflex C3, Avestin). The protein was purified with Ni-NTA agarose (Qiagen) under standard conditions. The tag was cleaved with tobacco etch virus protease and removed with a second Ni-NTA purification step. Subsequently, the protein was purified by size-exclusion chromatography on a HiLoad 26/600 Superdex 200 prep grade column (Cytiva) using ÄKTA start (Cytiva). Purified proteins were stored at −80 °C in a storage buffer (20 mM HEPES pH 8.0; 150 mM NaCl and 1 mM DTT).

### Reduction and desalting of purified proteins

Unless specified otherwise, purified proteins were reduced with freshly prepared DTT (10 mM) for 30 min at 4 °C. Excess DTT was removed by dual desalting with 0.5 ml Zeba Spin Desalting Columns (Thermo Fisher Scientific), preequilibrated with N_2_-purged assay buffer.

### Measurement of the roGFP2 redox state

Measurements were carried out in N_2_-purged 100 mM sodium phosphate buffer, pH 7.4, containing 100 μM diethylenetriaminepentaacetic acid, at 30 °C and 0.5% O_2_ in a CLARIOstar plate reader (BMG Labtech), using the top optics function (excitation at 405 and 485 nm, emission at 520 nm). Unless specified otherwise, prereduced roGFP2-containing proteins were dispensed at 100 nM final concentration and in 200 μl of final volume per well, in a black body, clear bottom Greiner F-bottom 96-well plate. The 405/520 and 488/520 fluorescence intensities were measured for 4 min before the addition of the test compound or corresponding vehicle. Fully oxidizing and fully reducing control conditions were applied at the end of each experiment, by adding 200 μM diamide and then 10 mM DTT. Competition assays were performed by adding freshly prepared compounds at least 15 min before the addition of 3-mercaptopyruvate. In the case of competing proteins, *S. cerevisiae* Uba4 and *H. sapiens* Trx1 were prereduced with DTT and desalted, as described above. Fatty acid free BSA was reduced as described previously^56^. In brief, 500 μM of freshly dissolved BSA was prereduced overnight with 50 mM beta-mercaptoethanol, at 4 °C. Excess beta-mercaptoethanol was removed by desalting three times with Zeba Spin Desalting Columns.

### Measurement of Tum1-dependent sulfur transfer to roGFP2 or SSP4

Here, 250 μl of streptavidin (SA) sepharose beads (50% slurry in storage buffer, 50 mM Tris-HCl, pH 8) were washed three times with 100 mM sodium phosphate buffer, pH 7.4, containing 100 μM diethylenetriaminepentaacetic acid. In all further steps the same phosphate buffer was used. All centrifugation steps were performed at 20*g* and 4 °C for 3 min. After the final washing step, SA beads were resuspended in 250 μl of phosphate buffer to obtain a 50% slurry. SA beads were then incubated with 125 μl of protein solution (13 μM SBP-His-SUMO-Tum1) for 1 h at 4 °C with gentle rotation. Beads were then incubated with DTT (10 mM final concentration) for 30 min at 4 °C. Then, beads were washed five times with 10 ml of phosphate buffer to remove DTT. After the final washing step, protein-saturated beads were resuspended in 250 μl of N_2_-purged phosphate buffer. For all further steps N_2_-purged phosphate buffer was used. For each reaction 25 μl of SA beads suspension was mixed with the reagents (as indicated in the corresponding figure legends) in a total volume of 100 μl. The samples were incubated for 5 min with gentle rotation at room temperature. Then the beads were spun down and 60 μl of supernatant was collected and further diluted with another 60 μl of buffer. The remaining beads were washed with 1 ml of phosphate buffer and resuspended again in 120 μl of buffer. Then, 10 μl of the resulting beads suspension or 10 μl of supernatant were added to the respective well in a 96-well plate (black body, clear bottom Greiner F-bottom 96) containing 420 nM roGFP2 or 100 μM SSP4 in 90 μl of buffer. Before use, roGFP2 was reduced and desalted as described above. For the competition experiment additional GSH was present in the well as indicated in the corresponding figure legend. roGFP2 and SSP4 fluorescence was recorded at 0.1% O_2_ and 30 °C with a CLARIOstar plate reader as described above. Following the reaction, diamide (200 μM) and DTT (10 mM) were added to determine the fully oxidized and reduced state.

### Electrode-based H_2_S measurements

Electrode measurements were carried out in a 100 mM sodium phosphate buffer, pH 7.4, at room temperature. The DTT-reduced and desalted Tum1-roGFP2 and Tum1(C259S)-roGFP2 fusions protein (2.5 μM) were mixed with 50 μM 3MP in a final volume of 1 ml (12-well Falcon plate, Corning). Detection of H_2_S was performed using the WPI Four-Channel Free Radical Analyzer with Lab-Trax 4/16 and an ISO-H_2_S-2 electrode. The concentration of released H_2_S was calculated from a previously determined Na_2_S standard curve.

### Thiocyanate measurement

Thiocyanate quantitation was based on the cyanolysis method of Wood^57^. The assay was carried out in 100 mM sodium phosphate, pH 7.4. Then 5 μM of DTT-reduced and doubly desalted Tum1-roGFP2 and Tum1(C259S)-roGFP2 proteins were mixed with 3,500 μM potassium cyanate. The mixture was preincubated at 30 °C for 5 min, and then incubated with 50 μM 3MP (final volume: 150 μl) at 30 °C for 10 min. The reaction was stopped by the addition of 150 μl of Goldstein’s reagent (61.88 mM ferric nitrate nonahydrate, 18.375% HNO_3_). The mixture was then centrifuged at 16,100*g* for 2 min and the supernatant transferred to clear 96-well plates. The product [Fe(SCN)(H_2_O)_5_]^2+^ was detected by measuring absorbance at 460 nm using a microplate reader (FLUOstar Omega, BMG Labtech). Thiocyanate levels were determined by use of a potassium thiocyanate standard curve.

### Yeast knockout library and transformation procedure

The BY4742 strain (*MATα his3Δ1 leu2Δ0 lys2Δ0 MET15 ura3Δ0*) was used in all experiments. Single-gene deletion strains were from the Euroscarf knockout library, generated using the KANMX marker^58^. Yeast strains BY4742 and Δ*tum1* were transformed with p415TEF or p416TEF plasmids with standard yeast transformation methods^59^. Transformants were selected at 30 °C on synthetically defined -Leu (for p415TEF) or -Leu-Ura (for p415TEF and p416TEF) (Formedium) agar plates, containing 1× yeast nitrogen base (BD Difco) and 2% glucose (Sigma-Aldrich).

### Measurement of the roGFP2 redox state in yeast cells

Yeast strains were grown in synthetically defined medium -Leu (for selection of p415TEF-based plasmids) or in synthetically defined medium -Leu -Ura (for selection of p415TEF and p416TEF-based plasmids) (Formedium), containing 1× yeast nitrogen base and 2% glucose. A single colony was used for inoculation and grown for roughly 20 h in 5 ml of growth medium at 30 °C under constant shaking. The following day, the culture was diluted to an OD of 0.25 and grown to OD 1.5 at 30 °C under constant shaking. Cells were collected by centrifugation at 4,000*g* for 10 min at 25 °C, washed with assay buffer (100 mM MES/Tris buffer, pH 6, 2% glucose) and then aliquoted in assay buffer at OD=4 (0.2 ml per well) in a black body, clear bottom Greiner F-bottom 96-well plate. Cells were then sedimented by centrifugation at 25*g* for 3 min at 25 °C. RoGFP2 fluorescence (excitation at 405 and 485 nm, emission at 520 nm) was measured with either a CLARIOstar or PHERAstar plate reader (BMG Labtech), at 30 °C, using the bottom optics option. The 405/520 and 488/520 fluorescence intensities were measured for 15 min before the addition of the testing compound or its corresponding vehicle. Yeast cells expressing an empty p415TEF plasmid were used as a background control to subtract autofluorescence. Fully reduced and fully oxidized controls were generated by adding 25 mM DTT and 20 mM diamide, respectively.

### Detection of persulfides by whole protein mass spectrometry

10 µM of reduced Tum1 was mixed with 10 µM of reduced Trx1(C35S), roGFP2, roGFP2(C148S) or roGFP2(C205S) in 100 mM sodium phosphate buffer, pH 7.4. The mixture was incubated at 30 °C for 5 min, before the addition of 60 µM 3MP and further incubation at 30 °C for 5 min. Resulting protein persulfides were alkylated by adding 1 mM monobromobimane (mBBr) (stock solution: 10 mM mBBr in 10% DMSO and 50 mM ammonium carbonate, pH 7.4) and incubating at room temperature and in the dark for 30 min. Excess mBBr was removed by twofold desalting with Zeba Spin 0.5 ml columns preequilibrated with 50 mM ammonium carbonate, pH 7.4. Samples were injected into a liquid chromatograph equipped with a POROS 10R1 column (Applied Biosystems) using 0.3% formic acid as the mobile phase. After 3 min, the mobile phase was switched to 50% of 0.3% formic acid and 50% of a 80% isopropanol/10% acetonitrile/0.3% formic acid mixture and held for 15 min. Mass spectra were obtained with a maXis electrospray ionization–time of flight mass spectrometer (Bruker Daltonik). Data were analyzed with Data Analysis v.4.2 (Bruker) and ESI Compass v.1.3.

### Depletion and overexpression of MPST

MPST was depleted in HEK293 MSR cells (GripTite, Thermo Fisher Scientific) using ON-TARGET plus small-interfering RNA SMARTpool (Dharmacon). The ON-TARGET plus nontargeting pool (Dharmacon D-001810-10-05) was used as a control. The siRNAs were transfected using DharmaFECT1 reagent (Dharmacon) as per the manufacturer’s instructions. For overexpression, HEK293 MSR cells were transfected with plasmids encoding roGFP2, MPST-roGFP2 or MPST(C248S)-roGFP2 using Lipofectamine 2000 (Thermo Fisher Scientific) following the manufacturer’s instructions. Protein expression levels were evaluated by immunoblotting, using anti-MPST (sc-374326) and anti-GFP (sc-9996) antibodies, both at 1:1,000 dilution.

### Fluorescent labeling of protein persulfides in mammalian cells

The dimedone switch method^1^ was used for relative quantitation of protein persulfides in mammalian cells with slight modifications. HEK293 MSR cells were grown to 80–90% confluency. Cells were lysed using cold HEN lysis buffer (50 mM HEPES, 1 mM EDTA, 0.1 mM neocuproine, 1% IGEPAL and 2% SDS; adjusted to pH 7.4) supplemented with protease inhibitor and 5 mM 4-chloro-7-nitrobenzofurazan (NBF-Cl). The lysate was then incubated at 37 °C for 30 min with occasional vortexing. The alkylated protein sample was precipitated with methanol/chloroform, as previously described^1^, and the resulting protein pellet was redissolved in 50 mM HEPES (pH 7.4) with SDS (2% final concentration). After adjusting the protein concentration to 3 mg ml^−1^, protein lysates were incubated with 50 μM Cy5-conjugated 4-(3-azidopropyl)cyclohexane-1,3-dione (DAz-2/Cy5) and subjected to copper(I)-catalyzed alkyne-azide cycloaddition at 37 °C for 30 min in the dark. The protein sample was again precipitated with methanol/chloroform, and the resulting pellet redissolved in HEPES with SDS (2% final concentration). The sample was then mixed with SDS loading buffer, boiled at 95 °C for 5 min, and loaded on a 12% acrylamide/bis-acrylamide gel (50 μg protein per lane). In-gel Cy5 fluorescence was recorded at 700 nm (LI-COR Odyssey Fc), followed by Coomassie staining to account for total protein content. Cy5 fluorescence and Coomasie staining intensity was quantified with ImageJ v.1.53p.

### Affinity purification of persulfidated proteins from mammalian cells

Affinity enrichment of persulfidated proteins from mammalian cells was based on the dimedone switch method^1^, with slight modifications. HEK293 MSR cells (MPST or mock depleted) were grown to 80–90% confluency in a 10 cm dish. Cells were lysed using cold HEN lysis buffer containing 5 mM NBF-Cl and the lysate was incubated for 30 min at 37 °C. Following methanol/chloroform precipitation, the protein pellet was dissolved in 50 mM HEPES buffer (pH 7.4) supplemented with 0.1% SDS. Preclearing of the protein solution (to remove endogenous biotinylated proteins) was performed by incubation with SA beads for 1 h. Following methanol/chloroform precipitation, the resulting protein pellet was redissolved in 50 mM HEPES buffer containing 2% SDS. The protein solution was then incubated with or without 50 μM DCP-Bio1 at 37 °C for 1 h, followed by methanol/chloroform precipitation. The protein pellet was redissolved in 50 mM HEPES buffer containing 0.1% SDS and the solution was incubated with SA beads at 4 °C overnight with agitation. The beads were washed three times with 50 mM HEPES buffer containing 0.001% Tween-20 and three times with plain 50 mM HEPES buffer. After washing, persulfidated proteins were eluted by incubation with 4 mM biotin for 30 min. The eluates were run on a SDS–PAGE gel and stained with Coomassie. Samples were then subjected to LC–tandem mass spectrometry (LC–MS/MS) analysis (below).

### LC–MS/MS analysis of affinity enriched persulfidated proteins

Following SDS–PAGE, four gel pieces per lane were manually excised. The gel pieces were washed once with 60 µl of 1:1 (v/v) 50 mM triethylammonium bicarbonate buffer (TEAB) and acetonitrile (ACN), pH 8.5 for 10 min and shrunk three times for 10 min each in 60 µl of ACN and washed in 60 µl of 50 mM TEAB, pH 8.5. Following reduction of proteins with 10 mM DTT in 100 mM TEAB at 57 °C for 30 min and dehydration of gel pieces, proteins were alkylated with 10 mM iodoacetamide in 100 mM TEAB at 25 °C for 20 min in the dark. Before protein digestion, gel pieces were washed with 60 µl of 100 mM TEAB and shrunk twice for 10 min in 60 µl of ACN. A total of 30 µl of 8 ng µl^−1^ in 50 mM TEAB trypsin solution (sequencing grade, Thermo Fisher Scientific) was added to the dry gel pieces and incubated 4 h at 37 °C. The reaction was quenched by addition of 20 µl of 0.1% trifluoroacetic acid (TFA). The resulting peptides were extracted once for 30 min with 30 µl 1:1 (v/v) 0.1% TFA and ACN, followed by gel dehydration with 20 µl ACN for 20 min, and washed with 30 µl of 100 mM TEAB for another 20 min. Finally, the gel was shrunk twice with 20 µl of ACN for 20 min. The supernatant from each extraction step was collected, concentrated in a vacuum centrifuge and dissolved in 15 µl 0.1% TFA. Nanoflow LC-MS2 analysis was carried out using an Ultimate 3000 LC system coupled to an Orbitrap QE HF (Thermo Fisher). An in-house packed analytical column (75 µm × 200 mm, 1.9 µm ReprosilPur-AQ 120 C18 material; Dr. Maisch) was used. Mobile phase solutions were prepared as follows, solvent A: 0.1% formic acid/1% acetonitrile, solvent B: 0.1% formic acid, 89.9% acetonitrile. Peptides were separated in a 25 min linear gradient from 3 to 23% B over 21 min and then to 38% B over 4 min, followed by washout with 95% B. The mass spectrometer was operated in data-dependent acquisition mode, automatically switching between MS and MS2. MS spectra (*m/z* 400–1,600) were acquired in the Orbitrap at 60,000 (*m/z* 400) resolution and MS2 spectra were generated for up to 15 precursors with normalized collision energy of 27 and isolation width of 1.4 *m/z*. The MS/MS spectra were searched against the Swiss-Prot H. sapiens protein database modified June 2020 and containing 20,531 sequences (UP000005640), and a customized contaminant database using Proteome Discoverer v.2.5 with Sequest HT (Thermo Fisher Scientific). A fragment ion mass tolerance was set to 0.2 Da and a parent ion mass tolerance to 10 ppm. Trypsin was specified as the enzyme. Following modifications of peptides were allowed: carbamidomethylation (cysteine), oxidation (methionine), deamidation (asparagine and glutamine), acetylation (protein N terminus), methionine loss (protein N terminus), NBF (163.0012, lysine and cysteine), DCP-Bio1 (394.1557, cysteine) and hydrolyzed DCP-Bio1 (168.0786, cysteine). Peptide quantification was done using a precursor ion quantifier node with summed abundances method set for protein abundance calculation. The mass spectrometry proteomics data have been deposited to the ProteomeXchange Consortium via the PRIDE partner repository with the dataset identifier PXD038309.

### Identification of MPST target proteins by LC–MS/MS data analysis

Common contaminants, proteins identified with less than two peptides, proteins not being a master protein within a protein group and proteins not containing cysteine residues were filtered out. This procedure generated a list of 322 quantified proteins. We only considered proteins that were at least twofold more abundant in the DCP-Bio1 treated sample compared to the nontreated control or were quantified in the DCP-Bio1 treated sample but did not reach the quantification threshold in the nontreated control. For the obtained 300 proteins, the ratio of protein abundance between MPST-depleted and wild-type samples was calculated and log_2_ transformed. Proteins depleted more than twofold in MPST-depleted cells were considered candidate MPST target proteins. The 64 most depleted candidate target proteins were queried against the STRING database^50^ considering only protein interactions for which there is experimental evidence with at least medium confidence. The network was edited in Cytoscape^51^. Protein subcellular localization was downloaded from ProteinAtlas^60^.

### Reporting summary

Further information on research design is available in the Nature Portfolio Reporting Summary linked to this article.

## Online content

Any methods, additional references, Nature Portfolio reporting summaries, source data, extended data, supplementary information, acknowledgements, peer review information; details of author contributions and competing interests; and statements of data and code availability are available at 10.1038/s41589-022-01244-8.

## Supplementary information

Supplementary Information Supplementary Tables 1–3.

Reporting Summary

## Data Availability

All data generated and analyzed in this study are included in this article. The mass spectrometry proteomics data have been deposited to the ProteomeXchange Consortium via the PRIDE partner repository with the dataset identifier PXD038309. Source data are provided with this paper.

## Source data

Source Data Fig. 1 roGFP2 degree of oxidation (OxD) values (Fig. 1a,b), Δintensity values (Fig. 1c), IC_50_ values (Fig. 1d,e).
Source Data Fig. 2 roGFP2 OxD values (Fig. 2a,b).
Source Data Fig. 3 roGFP2 OxD values (Fig. 3a,b).
Source Data Fig. 4 roGFP2 OxD values (Fig. 4a–d).
Source Data Fig. 5 Mass Spectrometry normalized peak intensity (Fig. 5a,b), roGFP2 OxD values (Fig. 5c–e).
Source Data Fig. 6 Relative Cy5 fluorescence (Fig. 6a,b), list of persulfidated proteins identified on mass spectrometry, along with their abundance in wild-type or MPST-depleted cells (Fig. 6c).
Source Data Extended Data Fig. 1 roGFP2 OxD values (Extended Data Fig. 1a), calculated H_2_S concentration values (Extended Data Fig. 1b), roGFP2 endpoint OxD values (Extended Data Fig. 1c,e,f) and calculated KCN concentration values (Extended Data Fig. 1d).
Source Data Extended Data Fig. 2 roGFP2 OxD values (Extended Data Fig. 2a–h).
Source Data Extended Data Fig. 3 roGFP2 OxD values (Extended Data Fig. 3a,b).
Source Data Extended Data Fig. 4 roGFP2 OxD values (Extended Data Fig. 4a).
Source Data Extended Data Fig. 5 Mass spectrometry normalized peak intensity (Extended Data Fig. 5a–d).
Source Data Extended Data Fig. 6 roGFP2 OxD values (Extended Data Fig. 6a–e).
Source Data Extended Data Fig. 7 Uncropped western blots.
