# Supplementary material for: 3-Mercaptopyruvate sulfur transferase is a protein persulfidase
Source: Nat Chem Biol. 2023 Feb 2;19(4):507–17. doi: 10.1038/s41589-022-01244-8 (PMC10060159; doi:10.1038/s41589-022-01244-8)
Supplement: Supplementary file 1 — Supplementary Tables 1–3. [file 41589_2022_1244_MOESM1_ESM.pdf]

nature chemical biology

Article

https://doi.org/10.1038/s41589-022-01244-8

3-Mercaptopyruvate sulfur transferase is a protein persulfidase

In the format provided by the authors and unedited

Supplementary Table 1: Reagents

**Reagent**	**Source**	**Identifier**
NEBuilder HiFi DNA Assembly Master Mix	New England Biolabs	E2621
Quikchange Site-Directed Mutagenesis Kit	Agilent	200519
Dithiothreitol	Panreac AppliChem	A2948
Zeba^{TM} Spin Desalting Columns	Thermo Scientific	89882, 87766
Sodium mercaptopyruvate dihydrate	Sigma-Aldrich	90374
3-mercaptolactic acid	Santa Cruz Biotechnology	sc-482537
L-cysteine hydrochloride monohydrate	Sigma-Aldrich	C7880
Sodium thiosulfate	Sigma-Aldrich	72049
Sodium sulfide	Sigma-Aldrich	407410
Potassium cyanide	Sigma-Aldrich	60178
Potassium thiocyanate	Sigma-Aldrich	P2713
Sodium sulfite	Sigma-Aldrich	S0505
Glutathione, reduced	Sigma-Aldrich	G6529
Beta-mercaptoethanol	Sigma-Aldrich	M3148
Bovine serum albumin, fatty acid free	Sigma-Aldrich	A8806
D-cysteine hydrochloride monohydrate	Sigma-Aldrich	C8005
D-alanine	Sigma-Aldrich	A7377
Monobromobimane	Sigma-Aldrich	B4380
4-chloro-7-nitrobenzofurazan	Sigma-Aldrich	163260
DAz-2	Cayman Chemicals	13382
Cyanine5 alkyne	Lumiprobe	B30B0
Copper(II)-TBTA complex	Lumiprobe	21050
Neocuproine	Sigma-Aldrich	N1501
IGEPAL	Sigma-Aldrich	I3021
Iodoacetamide	Sigma-Aldrich	I5161
Trifluoroacetic acid	Biosolve	202341
Acetonitrile	Carl-Roth	7330.1

Triethylammonium bicarbonate buffer	Sigma-Aldrich	T7408
Ni^{2+}-Sepharose beads	Cytiva	17526801
PreScission Protease	Cytiva	27084301
GST-sepharose	Cytiva	17527901
Streptavidin (SA) sepharose beads	Cytiva	17511301

Supplementary Table 2: Plasmids

Plasmid	Reference
pQE60-roGFP2-His	[1]
pETHisSUMO-Tum1-roGFP2	This work
pETHisSUMO-Tum1(C259S)-roGFP2	This work
pETHisSUMO-Tum1	This work
pETHisSUMO-Tum1(C259S)	This work
pET-SBP-HisSUMO-Tum1	This work
p415TEF-roGFP2	[2]
p415TEF-Tum1-roGFP2	This work
p415TEF-Tum1(C259S)roGFP2	This work
p415TEF-Su9roGFP2	This work
p415TEF-Su9Tum1-roGFP2	This work
p415TEF-Su9Tum1(C259S)-roGFP2	This work
p416TEF-Su9DAAO	This work
pLPCx roGFP2	This work
pLPCx MPST-roGFP2	This work
pLPCx MPST(C248S)-roGFP2	This work

Supplementary Table 3: Primers

Name	Sequence	Purpose
p415TEF(NcoI)-Tum1	AAGTTTTCTAGAGGATCCACATGCCATTATTTGATCTTATTTC	Amplify Tum1 to assemble with p415TEF(NcoI digested) and linker-roGFP2
linker.roGFP2-Tum1	CACCACCTGAACCACCATCTCTGTTTTCAGCAATC	Amplify Tum1 to assemble with p415TEF(NcoI digested) and linker-roGFP2
Tum1-linker.roGFP2	AAACAGAGATGGTGGTTCAGGTGGTGGTGGTTCAGGTGGTGGTGGTTCAGGTGGAGGAGGATCAGGAGGAGGAGGATCAGGAGGAGGAGGATCAGGAGGAGCTAGCGAATTCTCAAAG	Amplify linker-roGFP2 to assemble with Tum1 and p415TEF (HindIII digested)
p415TEF(HindIII)-linker.roGFP2	TGACTCGAGGTCGACGGTATCGATATTATTTATACAATTCATCCATACCC	Amplify linker-roGFP2 to assemble with Tum1 and p415TEF (HindIII digested)
Tum1_C259S_fw	CCAACTATTTGCTCTTCTGGAACTGGCGTTTCAGG	Site-directed mutagenesis of Tum1 catalytic Cys 259 to serine
Tum1_C259S_rev	CCTGAAACGCCAGTTCCAGAAGAGCAAATAGTTGG	Site-directed mutagenesis of Tum1 catalytic Cys 259 to serine
p415TEFSu9(BamHI)-Tum1-roGFP2	GAAGCGCGCCTACTCTTCCGGATCCCCATTATTTGATCTTATTTCTCC	Amplify Tum1-roGFP2 to assemble with p415TEF + Su9 signal peptide for mitochondrial localization (BamHI/HindIII digested)
p415TEFSu9(HindIII)-Tum1-roGFP2	CGAGGTCGACGGTATCGATATTATTTATACAATTCATCCATACC	Amplify Tum1-roGFP2 to assemble with p415TEF + Su9 signal peptide for mitochondrial localization (BamHI/HindIII digested)
p416TEFSu9(BamHI)-Su9	TAAGTTTTCTAGAACTAGTGGATCCATGGCCTCCACTCGTGTC	Amplify F_0-ATPase (Su9) from *Neurospora crassa* to assemble with p416TEF (BamHI/HindIII digested) and DAAO.
DAAO-Su9	TCTGGCTGTGGGATCCGGAAGAGTAGGC	Amplify F_0-ATPase (Su9) from *Neurospora crassa* to assemble with p416TEF (BamHI/HindIII digested) and DAAO.

Su9-DAAO	TTCCGGATCCCACAGCCAGAAGAGGGTG	Amplify DAAO to assemble with p416TEF (BamHI/HindIII digested) and F_0-ATPase (Su9) from *N. crassa*.
p416TEFSu9(HindIII)-DAAO	CGAGGTCGACGGTATCGATAAGCTTTTAGCTCTCCCTAGCTGC	Amplify DAAO to assemble with p416TEF (BamHI/HindIII digested) and F_0-ATPase (Su9) from *N. crassa*.
pETHisSUMO_fw	AGACAAGCTTAGGTATTTATTCG	Amplify linearized pETHisSUMO
pETHisSUMO-rv	TGGACCCTGGAACAGAAC	Amplify linearized pETHisSUMO
pETHisSUMO-Tum1-roGFP2-fw	AAGTTCTGTTCCAGGGTCCAATGCCATTATTTGATCTTATTTC	Amplify Tum1-roGFP2 to assemble with linearized pETHisSUMO vector
pETHisSUMO-Tum1-roGFP2-rv	ATAAATACCTAAGCTTGTCTTTATTTATACAATTCATCCATACCC	Amplify Tum1-roGFP2 to assemble with linearized pETHisSUMO vector
roGFP2_C148S_fw	GGAGTACAACTACAACAGCCACAACGTCTATATC	Mutagenesis cys148 from roGFP2-His expressed in pQE60 vector to serine
roGFP2_C148S_rv	GATATAGACGTTGTGGCTGTTGTAGTTGTACTCC	Mutagenesis cys148 from roGFP2-His expressed in pQE60 vector to serine
roGFP2_C205S_fw	ACCACTACCTGAGCACCAGCTCCGCCCTGAGCAAA	Mutagenesis cys205 from roGFP2-His expressed in pQE60 vector to serine
roGFP2_C205S_rv	TTTGCTCAGGGCGGAGCTGGTGCTCAGGTAGTGGT	Mutagenesis cys205 from roGFP2-His expressed in pQE60 vector to serine
pETHisSUMO-Tum1_fw	AAGTTCTGTTCCAGGGTCCAATGCCATTATTTGATCTTATTTC	Amplify Tum1 (no roGFP2) to anneal to linearized pETHisSUMO
pETHisSUMO-Tum1_rv	ATAAATACCTAAGCTTGTCTTTAATCTCTGTTTTCAGCAATC	Amplify Tum1 (no roGFP2) to anneal to linearized pETHisSUMO
MPST_C248S_fw	CTGGTTGCTACAAGCGGTTCAGGCG	Mutagenesis cys248 from human MPST to serine
MPST_C248S_rv	CGCCTGAACCGCTTGTAGCAACCAG	Mutagenesis cys248 from human MPST to serine

Supplementary references

1. Gutscher, M. *et al.* Real-time imaging of the intracellular glutathione redox potential. *Nat Methods* 5, 553–559 (2008)

2. Morgan, B. *et al.* Multiple glutathione disulfide removal pathways mediate cytosolic redox homeostasis. *Nat Chem Biol* 9, 119–125 (2013)
