# Supplementary figures and images for: 3-Mercaptopyruvate sulfur transferase is a protein persulfidase
Source: Nat Chem Biol. 2023 Feb 2;19(4):507–17. doi: 10.1038/s41589-022-01244-8 (PMC10060159; doi:10.1038/s41589-022-01244-8)

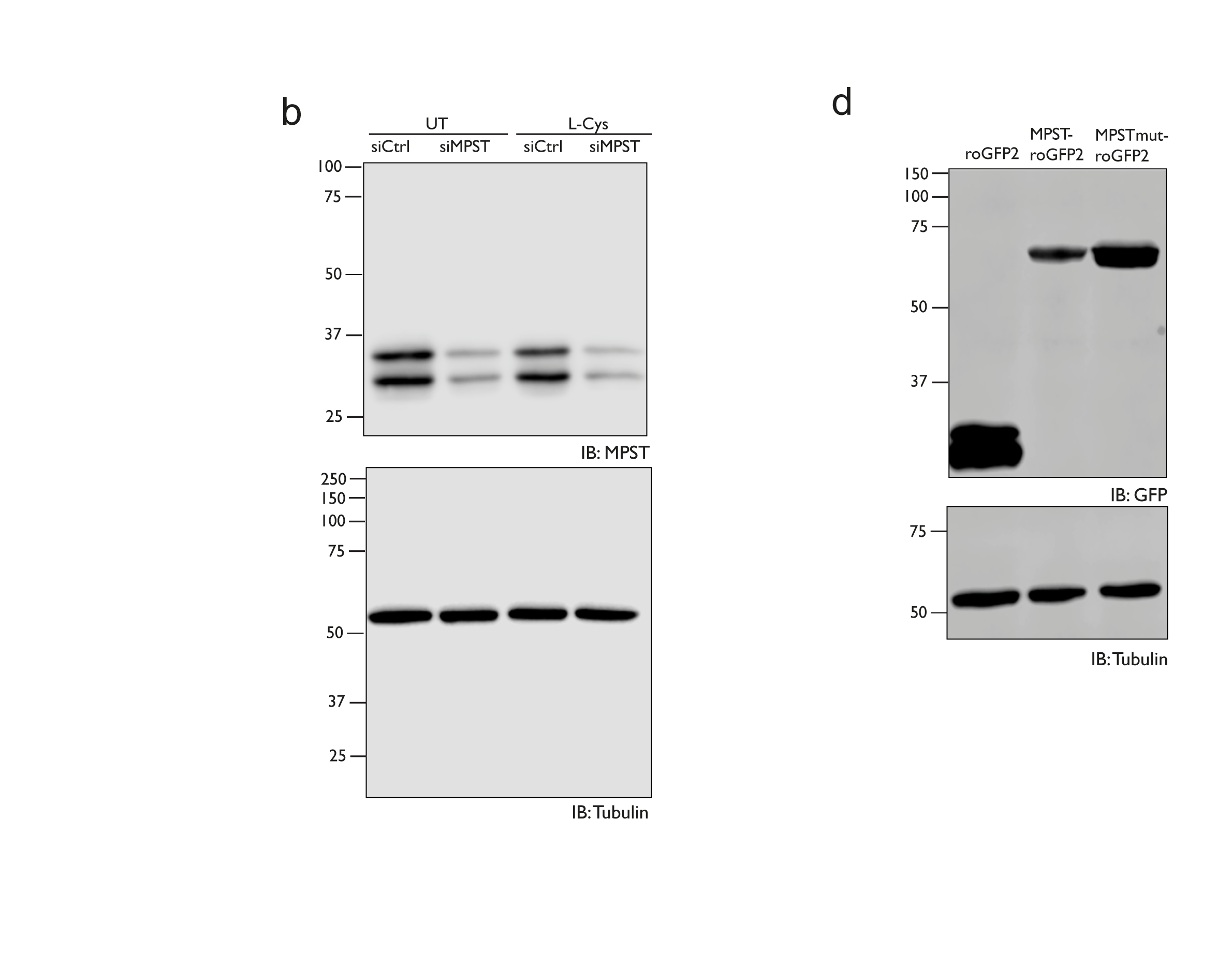

Supplement: Source Data Extended Data Fig. 7 — Uncropped western blots. [file 41589_2022_1244_MOESM15_ESM.tif]
